# Dynamic proteome trade-offs regulate bacterial cell size and growth in fluctuating nutrient environments

**DOI:** 10.1038/s42003-023-04865-4

**Published:** 2023-05-05

**Authors:** Josiah C. Kratz, Shiladitya Banerjee

**Affiliations:** 1 Department of Biological Sciences, Carnegie Mellon University, Pittsburgh, PA 15213 USA; 2 Department of Physics, Carnegie Mellon University, Pittsburgh, PA 15213 USA

**Keywords:** Computational biophysics, Numerical simulations, Bacterial systems biology

## Abstract

Bacteria dynamically regulate cell size and growth to thrive in changing environments. While previous studies have characterized bacterial growth physiology at steady-state, a quantitative understanding of bacterial physiology in time-varying environments is lacking. Here we develop a quantitative theory connecting bacterial growth and division rates to proteome allocation in time-varying nutrient environments. In such environments, cell size and growth are regulated by trade-offs between prioritization of biomass accumulation or division, resulting in decoupling of single-cell growth rate from population growth rate. Specifically, bacteria transiently prioritize biomass accumulation over production of division machinery during nutrient upshifts, while prioritizing division over growth during downshifts. When subjected to pulsatile nutrient concentration, we find that bacteria exhibit a transient memory of previous metabolic states due to the slow dynamics of proteome reallocation. This allows for faster adaptation to previously seen environments and results in division control which is dependent on the time-profile of fluctuations.

## Abstract (web summary)

A quantitative theory connects bacterial growth and division rates to proteome allocation and cell size control in time-varying nutrient environments.

## Introduction

In their natural environment, bacteria must be able to sense and adapt rapidly to time-varying environmental stressors to survive and proliferate. Not surprisingly, bacteria exhibit tight regulatory control over their growth physiology and cell morphology^1,2^, and alter both in response to fluctuating nutrient perturbations, resulting in dynamic growth rate and cell size changes in time-varying environments^3–6^.

Significant research has gone into understanding how bacterial cell size is coupled to growth rate^7^, DNA replication^8,9^, and gene expression^10^ at steady-state, and how size homeostasis is maintained despite division and growth rate noise^11,12^. In addition, characterization of a large portion of the steady-state bacterial proteome across different growth conditions has improved understanding of the resource allocation strategies employed by bacteria in different environments^13–15^. Motivated by experimental data, various coarse-grained models of cell physiology have been developed in recent years, which explain the regulation of cellular growth rate and cell size control from underlying proteome allocation strategies at steady-state^10,16–20^. However, bacteria do not exist naturally in such conditions, but instead thrive in rapidly changing environments. As a result, it remains unclear how cells sense changes in the environment and dynamically regulate division and growth in response.

Bacteria reallocate their proteome to relieve metabolic or translational bottlenecks and increase growth rate under a given nutrient limitation^21^, but do not always allocate resources in order to optimize steady-state growth rate^22^. For example, bacteria maintain a fraction of inactive ribosomes at steady state regardless of nutrient condition, presumably as a reserve which can be deployed to quickly increase growth rate during nutrient upshift^4,23^. This apparent strategy highlights the challenges of resource allocation in dynamic environments, specifically that organisms must weigh the trade-offs between optimizing growth rate at steady-state and employing mechanisms that are costly at steady-state but that hasten adaptation to environmental changes^4,24^. In addition, the molecular mechanisms connecting dynamic resource allocation to division control in bacteria are not clear, nor is our understanding of how these allocation strategies are affected by the temporal pattern of environmental fluctuations. Furthermore, it is unclear if bacterial size modulation is simply a byproduct of the complex cellular response to changing environmental conditions, or if it serves as an adaptive mechanism employed by the cell to improve fitness in time-varying environments.

To understand the dynamics of bacterial growth physiology and size control in dynamic nutrient environments, we have developed a coarse-grained proteome sector model which connects gene expression to growth rate and division control, and accurately predicts the cell-level *E. coli* response to nutrient perturbations in both exponential and stationary phase seen in experimental data^5,25^. This is done by integrating the dynamics of biochemical elements with decision-making rules for cell division. Motivated by recent experimental work which suggests that division is regulated independently of DNA replication^26^, we employed a chromosome-independent threshold accumulation model to determine division timing. We applied this model to study how cells allocate intracellular resources dynamically in response to time-varying nutrient conditions, and found that growth rate and cell size control is governed by dynamic trade-offs between biomass accumulation and cell division. Specifically, our model predicts that bacteria temporarily divert resources to ribosome production over division protein production during nutrient upshift, resulting in a transient delay in division and an overshoot in added cell volume per generation as cells prioritize biomass accumulation. Conversely, in response to nutrient downshift, cells prioritize division over growth, resulting in a rapid decrease in added volume and interdivision time before relaxing to their steady-state values. As a result, population and single-cell growth rates decouple outside of steady-state, potentially serving as an adaptive mechanism in time-varying environments. Lastly, when simulating pulsatile nutrient conditions, we find that growth rate and cell size recovery time after pulse cessation both increase with increasing pulse duration. Our model suggests that this transient memory of previous environments is a result of the slow dynamics of proteome reallocation, and provides a passive mechanism for faster adaptation in fluctuating environments.

## Results

### Dynamic proteome allocation in time-varying nutrient environments

**Fig. 1:**
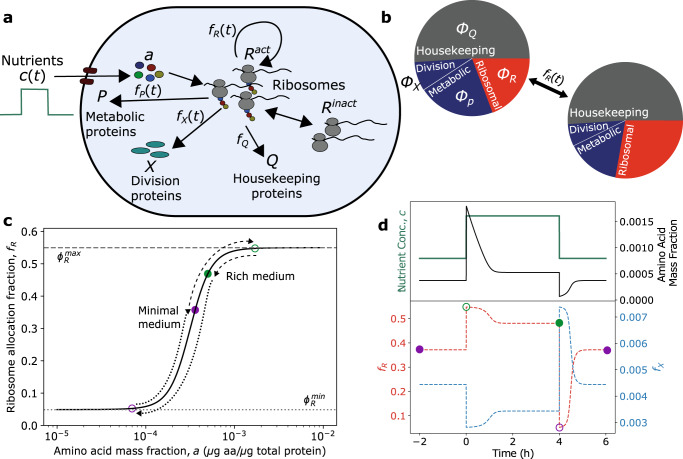
Dynamic resource allocation model for cell growth and division control in dynamic environments. **a** Schematic of coarse-grained model of bacterial cell size control and growth physiology. Nutrients (*c*) are imported by metabolic proteins (*P*) and converted to amino acids (*a*), which are then consumed by ribosomes (*R*) to produce proteins. Division occurs once a threshold amount of division proteins (*X*) have been accumulated. **b** By dynamically regulating the fraction of the total translational flux devoted to each proteome sector *i*, *f*_*i*_(*a*(*t*)), in response to changes in *a* triggered by environmental changes, the cell alters its proteome composition, and thus its size and growth rate. **c** The dependence of *f*_*R*_ on *a* is the given by their steady-state relationship. The path of *f*_*R*_ in response to a nutrient-rich pulse is shown with colored circles corresponding to the timepoints shown in **d**. *f*_*R*_ is initially given by its steady-state value in minimal medium (purple, closed). A shift to rich medium results in a transient increase in *f*_*R*_ close to its maximum value (green, open), before relaxing back to its new steady-state value (green, closed). The path during upshift is given by the dashed line. A shift back to minimal medium results in a temporary drop in *f*_*R*_ close to its minimum value (purple, open), before relaxing back to the original steady-state value (purple, closed). The path during downshift is given by the dotted line. **d** Representative dynamics of amino acid mass fraction (top) and proteome allocation fractions (bottom) during a nutrient pulse. See Table 1 for a list of parameters.

Bacterial cells integrate time-varying environmental information through a complex set of regulatory networks to control gene expression. Despite this complexity, steady-state proteomics reveals that the expression of proteins with similar function are regulated reciprocally in response to growth rate perturbations, such that various proteome sectors can be defined which coarse-grain the cellular milieu into a limited number of collective state variables^13–15^. To investigate *E. coli* cell size and growth rate control in time-varying nutrient environments, we developed a dynamic model which partitions the proteome into four sectors: ribosomal, metabolic, division, and “housekeeping” (Fig. 1a). In this framework, the environment contains time-varying nutrients with concentration *c*, which the cell imports and converts into amino acids using metabolic proteins with protein mass fraction *ϕ*_*P*_. The kinetics of protein translation are limited by the abundance of multiple metabolites, the specific identity of which can change with time and nutrient environment. As a result, we define *a* as the mass fraction of the coarse-grained growth-limiting amino acid pool. This pool is consumed by translating ribosomes, with mass fraction *ϕ*_*R*_, to synthesize each of the four proteome sectors. As a result, the ribosome mass fraction sets the exponential growth rate, $\kappa ={{{{{{{\rm{d}}}}}}}}\ln M/{{{{{{{\rm{d}}}}}}}}t={{{{{{{\rm{d}}}}}}}}\ln V/{{{{{{{\rm{d}}}}}}}}t$, given by

1 $$\kappa ={\kappa }_{t}(a)\left({\phi }_{R}-{\phi }_{R}^{\min }\right)-{\mu }_{{{{{{{{\rm{ns}}}}}}}}},$$

where *μ*_ns_ is the non-specific degradation rate constant, ${\phi }_{R}^{\,{{{\min}}}\,}$ denotes the fraction of ribosomes which are not actively engaged in translation, and *κ*_*t*_(*a*) is the translational efficiency, which is dependent on amino acid availability such that translation becomes significantly attenuated at low intracellular amino acid levels (see “Methods”).

**Table 1:** Model parameters.

Parameter	Description	Value	Growth condition	Figure number
${\phi }_{R}^{\min }$	Inactive ribosome fraction^16^	0.049	All	All
${\phi }_{R}^{\max }$	Maximum flux allocation to ribosome production^16^	0.55	All	All
*a*_*t*_	Translation attenuation threshold^28^	10^−4^	All	All
*a*_*n*_	Feedback inhibition threshold^28^	10^−3^	All	All
${\kappa }_{t}^{0}$ (h^−1^)	Translational efficiency rate constant	2.6	All	1, 3–6
		4.8	All	2
${\kappa }_{n,low}^{0}$ (h^−1^)	Nutritional efficiency rate constant in nutrient-poor media	4.8	Exponential	1, 3, 4, 6
		0	Stationary	5
${\kappa }_{n,high}^{0}$ (h^−1^)	Nutritional efficiency rate constant in nutrient-rich media	10	All	1
		60	All	3–6
*μ*_ns_ (h^−1^)	Nonspecific degradation rate	0	Exponential	2–4, 6
		0.1	Stationary	5
*μ*_*X*_ (h^−1^)	Division protein degradation rate, strain specific	2.5	All	3–6
		0.1	All	2
*γα* (μm^−3^)	Strain-specific parameter, represents contribution to *k*_*P*_ from co-regulated portion of *f*_*X*_	4.5	All	1, 3–6
		3.6	All	2
*γβ* (μm^−3^)	Strain-specific parameter, represents contribution to *k*_*P*_ from basal allocation fraction of *f*_*X*_	1.1	All	1, 3–6
		0.34	All	2

See Supplementary Information for more details.

In response to changes in nutrient availability, the cell reallocates its proteome by altering the fraction of translational flux, ${J}_{t}(t)={\kappa }_{t}({\phi }_{R}(t)-{\phi }_{R}^{\,{{{\min}}}\,})$, devoted to each sector, such that the dynamics of each sector can be written as

2 $$\frac{{{{{{{{\rm{d}}}}}}}}}{{{{{{{{\rm{d}}}}}}}}t}{{{{{{{\boldsymbol{\phi }}}}}}}}(t)={J}_{t}(t)({{{{{{{\boldsymbol{f}}}}}}}}(t)-{{{{{{{\boldsymbol{\phi }}}}}}}}(t)),$$

where the vectors ***ϕ***(*t*) = [*ϕ*_*R*_(*t*), *ϕ*_*P*_(*t*), *ϕ*_*X*_(*t*), *ϕ*_*Q*_(*t*)] and ***f***(*t*) = [*f*_*R*_(*t*), *f*_*P*_(*t*), *f*_*X*_(*t*), *f*_*Q*_(*t*)] denote the protein mass fraction and translational flux allocation fraction of each sector at time *t*, respectively. *E. coli* proteomics data reveal that a significant fraction of the proteome is invariant to environmental perturbations^13^. As a result, we define the housekeeping sector such that it contains all the proteins whose proteome allocation is not growth rate dependent. Consequently, *ϕ*_*Q*_ = *f*_*Q*_ = constant, and *f*_*R*_(*t*) + *f*_*P*_(*t*) + *f*_*X*_(*t*) = 1 − *f*_*Q*_ = *ϕ*^max^.

To model division control, we employ a threshold accumulation model of cell division in which division is triggered after a cell accumulates a fixed amount of division proteins^10,17,26,27^. Since the total protein abundance per cell scales with growth rate^7,8^ and if the threshold remains constant^5,26^, the average protein mass fraction of division proteins per cell necessarily decreases to maintain the constancy of the threshold, and thus must be part of the metabolic sector^12,17^. Consequently, we assume that allocation to the division sector, *f*_*X*_(*t*), is given by a linear combination of a basal allocation fraction, *β*, and a time-dependent fraction whose expression is co-regulated as part of a larger metabolic sector, ${f}_{P}^{* }(t)$. As a result, the flux allocation constraint can be simplified such that ${f}_{R}(t)+{f}_{P}^{* }(t)={\phi }_{R}^{\,{{{\max}}}\,}$, where ${\phi }_{R}^{\,{{{\max}}}}={\phi }^{{{{\max}}}}-\beta$ represents the upper limit to the allocation fraction devoted to ribosomal proteins. Using the simplified constraint, *f*_*X*_(*t*) can be expressed such that its time dependence is solely through *f*_*R*_(*t*), yielding

3 $${f}_{X}(t)=\alpha \left({\phi }_{R}^{\max }-{f}_{R}(t)\right)+\beta ,$$

where *α* is the fraction of the co-regulated sector ${f}_{P}^{* }(t)$ made up of division proteins. From Eq. (3), we see that when the fraction of cellular resources allocated to ribosome production increases, metabolic and division protein translational flux is necessarily downregulated, and vice versa (Fig. 1b). This prediction is supported by *E. coli* proteomics data^15^ (Supplementary Fig. 1), and highlights a trade-off that cells must make between biomass accumulation and division in dynamic environments.

Critically, as all other proteome sectors are defined in terms of *f*_*R*_(*t*), the time-dependence of *f*_*R*_ must be specified. To do so, we assume that dynamic reallocation is driven by gene-regulatory networks which are dependent on the amino acid pool, such that the time dependence of *f*_*R*_ is given through its dependence on the time-varying amino acid mass fraction *a*, thus *f*_*R*_(*t*) = *f*_*R*_(*a*(*t*)). In this way *a* serves as our key kinetic variable, as opposed to defining gene expression dynamics in terms of the translational activity like a model by Erickson et al.^18^. The dynamics of *a* are coupled to Eq. (2) and are given by the difference in the metabolic and translational fluxes, such that d*a*/d*t* = *J*_*n*_ − *J*_*t*_, where the metabolic flux, *J*_*n*_, is proportional to the metabolic sector mass fraction, *ϕ*_*P*_. The dynamics of *a* can be written in terms of the proteome mass fractions as

4 $$\frac{{{{{{{{\rm{d}}}}}}}}a}{{{{{{{{\rm{d}}}}}}}}t}={\kappa }_{n}(a)({\phi }^{{{{\max}}}}-{\phi }_{R}-{\phi }_{X})+{\mu }_{{{{{{{{\rm{ns}}}}}}}}}-{\kappa }_{t}(a)\left({\phi }_{R}-{\phi }_{R}^{\,{{{\min}}}\,}\right),$$

where *κ*_*n*_(*a*) is the nutritional efficiency (see “Methods”), which becomes significantly attenuated at high values of *a* to reflect end-product inhibition of biosynthesis pathways and inactivation of nutrient importers at high intracellular amino acid concentrations^28^.

Changes in environmental nutrient availability result in a flux imbalance which alters the size of the amino acid pool. In this way, *a* acts as a read-out of flux imbalance, and so by altering proteome allocation in response to *a*, the cell can dynamically respond to nutrient changes. To obtain the functional form of *f*_*R*_(*a*(*t*)), we assume that *a*(*t*) sets the allocation fraction *f*_*R*_(*a*(*t*)) via the steady-state relation ${f}_{R}^{* }(a)$, such that ${f}_{R}(a(t))={f}_{R}^{* }(a(t))$. Furthermore, we assume that the cell maximizes translational flux at steady-state, which allows us to express *f*_*R*_ solely in terms of the amino acid mass fraction, *a* (see Methods and Supplementary Notes 1 and 2). ${f}_{R}^{* }(a)$ is shown graphically in Fig. 1c, and predicts that proteome allocation is altered to reduce growth bottlenecks. Namely, when *a* is high, ribosome synthesis is prioritized in order to increase translation flux, but when *a* is low, metabolic protein synthesis is prioritized to increase nutrient import. Mathematically, any monotonically increasing function for *f*_*R*_(*a*) will produce this type of regulatory behavior. However, by choosing *f*_*R*_(*a*) to be given by ${f}_{R}^{* }(a)$, we also ensure that translational flux is maximized at steady state. This assumption of growth-rate maximization at steady-state has proved fruitful in previous theoretical models to explain bacterial growth laws^16,28–32^, and has been observed experimentally to be the case for many nutrient-limiting conditions^22^. Furthermore, it has been experimentally observed that *E. coli* cells evolve their metabolism towards a state that maximizes growth rate^33–35^.

Using the above framework, the dynamics of proteome allocation can be simulated in time-varying nutrient environments by numerically solving the coupled Eqs. (2) and (4) (Fig. 1d). In response to a pulse of nutrients, allocation to ribosome synthesis increases drastically to its maximum value before slowly relaxing to its steady-state value in rich media (Fig. 1d). In contrast, allocation to division protein synthesis drops significantly before slowly relaxing to a lower steady-state value. Following cessation of the nutrient-rich pulse, the opposite trends occur for each allocation fraction, resulting in an overshoot in *f*_*X*_ and undershoot in *f*_*R*_ before both returning to their initial values (Fig. 1d).

At the molecular level, this regulation of gene expression is carried out by the signaling molecule guanosine tetraphosphate (ppGpp), which is synthesized when charged tRNA levels are low^36,37^. As charged tRNA abundance is proportional to amino acid levels, ppGpp thus indirectly acts as a sensor of the amino acid pool. In response to decreased amino acid levels, ppGpp levels increase and repress rRNA expression^36,37^. Conversely, when amino acids are abundant, ppGpp levels decrease to promote ribosome production. ppGpp production also induces genome-wide changes in protein expression. Experimental results have shown that ppGpp activates metabolic and division protein expression^38^, and when overexpressed decreases cell size^39^. In this way, ppGpp acts as a global regulator which modulates expression of the three dynamic proteome sectors, and thus regulates translational flux, by responding to changes in amino acid concentration.

### Growth-rate dependent increase in cell size arises from trade-off between biomass accumulation and division protein synthesis

To predict cell size control behavior, the dynamics of proteome allocation must be connected to the dynamics of the number of accumulated division proteins per cell after birth, *X*(*t*), where cells divide at *t* = *τ* after accumulating a fixed number of X proteins, *X*(*τ*) = *X*_0_. Using the relation *X* = *ϕ*_*X*_*Vρ*_*c*_/*m*_*X*_ − X^*^, where *ρ*_*c*_ is the protein mass density of the cell, *m*_*X*_ is the mass of division molecule X, and X^*^ is the amount of division proteins at birth, the dynamics of *ϕ*_*X*_ can be used to identify the dynamics of the fraction of the accumulated number of division proteins required to trigger cell division, $\tilde{X}=X/{X}_{0}$,

5 $$\frac{{{{{{{{\rm{d}}}}}}}}\tilde{X}}{{{{{{{{\rm{d}}}}}}}}t}=\gamma {f}_{X}{J}_{t}V-{\mu }_{X}\tilde{X},$$

where *γ* = *ρ*_*c*_/*X*_0_*m*_*X*_ and *μ*_*X*_ is the degradation rate of division proteins. We thus identify the division protein synthesis rate per unit volume as *k*_*P*_(*t*) = *γf*_*X*_(*t*)*J*_*t*_(*t*). By numerically solving proteome allocation and volume dynamics in conjunction with the division rules given by Eq. (5), single cell size and growth rate dynamics can be simulated in fluctuating nutrient environments.

**Fig. 2:**
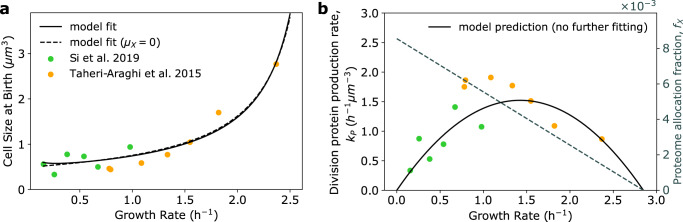
Growth-rate dependent trade-off between biomass accumulation and division protein synthesis sets steady-state bacterial cell size. **a** Steady-state relationship between population average cell size at birth and growth rate for *E. coli*. Dotted line shows best fit of Eq. (7), yielding parameters *γα*, *γβ*, and *κ*_*t*_. Solid line shows best fit with inclusion of degradation rate *μ*_*X*_, parameters are given in Table 1. Experimental data are of *E. coli* K-12 NCM3722 cells from refs. ^12, 26^. **b** Non-monotonic dependence of the division protein production rate, *k*_*P*_, on growth rate, where *k*_*P*_ is estimated from experimental data as 〈*κ*〉/〈*V*_0_〉. The model predicts the non-monotonic dependence (solid line, parameters given by best fit from **a**) despite a linear decrease in allocation fraction to the division protein sector with increasing growth rate (dotted line).

To test the validity of our resource allocation model, we first examined if the model can reproduce experimentally observed steady-state physiological behaviors of bacterial cells, in particular the increase in average cell size with growth rate under nutrient perturbations (Fig. 2a)^1,7,8^. At steady-state, *f*_*R*_ = *ϕ*_*R*_ (Eq. (2)), allowing the rate of division protein synthesis *k*_*P*_ to be written solely as a function of growth rate. In moderate to fast exponential growth conditions, the effects of protein degradation are negligible. Thus assuming *κ* ≫ *μ*_ns_, we arrive at

6 $${k}_{P}(\kappa )=\gamma (\alpha (\Delta \phi -\kappa /{\kappa }_{t})+\beta )\kappa ,$$

where $\Delta \phi ={\phi }_{R}^{\max }-{\phi }_{R}^{\min }$. When *κ* ≫ *μ*_*X*_, this model recapitulates the adder principle employed by *E. coli* to achieve size homeostasis^12^, in which a constant amount of volume, Δ, is added each generation irrespective of birth size, Δ ≈ *V*_0_ ≈ *κ*/*k*_*P*_. We discuss deviations from this size control behavior in slow growth conditions, when degradation effects become important, in the last Results section. Substituting Eq. (6) into the expression for birth size yields a novel formulation of the size law^7^, which links nutrient-limited growth rate to cell size (Fig. 2a), such that

7 $${V}_{0}(\kappa )=\frac{1}{\gamma \alpha (\Delta \phi -\kappa /{\kappa }_{t})+\gamma \beta }.$$

The above equation can be fit well to experimental data^8,12^ (Fig. 2a), to predict the cell type-specific or strain-specific parameters *γα*, *γβ*, and *κ*_*t*_. *f*_*X*_ can then be inferred from these parameters by assuming the identity of X proteins (see Supplementary Note 3). The data can also be fit well by the full model which includes degradation (Eq. (S.6) in Supplementary Note 4). We found that including the effects of X protein degradation are essential to quantitatively capturing dynamic size control (Supplementary Fig. 2), and so we use best fit parameters from the full model to numerically predict the dependence of *k*_*P*_ on *κ* (Fig. 2b). Interestingly, Eq. (6) predicts a non-monotonic dependence of the division protein production rate on growth rate. This behavior can be understood by considering the effects of both *f*_*X*_ and *J*_*t*_, where here *J*_*t*_ = *κ* when nonspecific degradation effects are negligible. As growth rate decreases, translational flux allocation to division protein production, *f*_*X*_, increases while overall translational flux, *J*_*t*_, decreases (Fig. 2b). As such, at fast growth rates, decreasing *κ* results in an increase in *k*_*P*_ due to an increase in *f*_*X*_. Conversely, at slow growth rates this increase in *f*_*X*_ is dominated by the decrease in *J*_*t*_, resulting in a reduction in *k*_*P*_. At intermediate growth rates translational flux and allocation are simultaneously moderately high, consequently yielding the maximum *k*_*P*_ value.

The expression for cell volume given in Eq. (7) predicts a maximum growth rate given by ${\kappa }_{\max }={\kappa }_{t}(\Delta \phi +\beta /\alpha )$. This theoretical maximum, however, is nonphysical as it assumes that *f*_*X*_ = *ϕ*_*X*_ = 0, which is never the case (Eq. (3)). Growth rate is maximum when ${\phi }_{R}={\phi }_{R}^{\max }$, thus giving an upper limit to the physical growth rate at ${\kappa }_{\max }={\kappa }_{t}\Delta \phi$. Eq. (7) also implies that there is no bound on cell size. However, our expression for *f*_*X*_ constrains cell size to a finite value. When allocation to ribosomes is maximal, *ϕ*_*X*_ = *β*, such that the maximum birth volume *V*_0_ is given by ${V}_{0}^{\max }=1/\gamma \beta$.

### Cells transiently prioritize biomass accumulation over division during nutrient upshifts

**Fig. 3:**
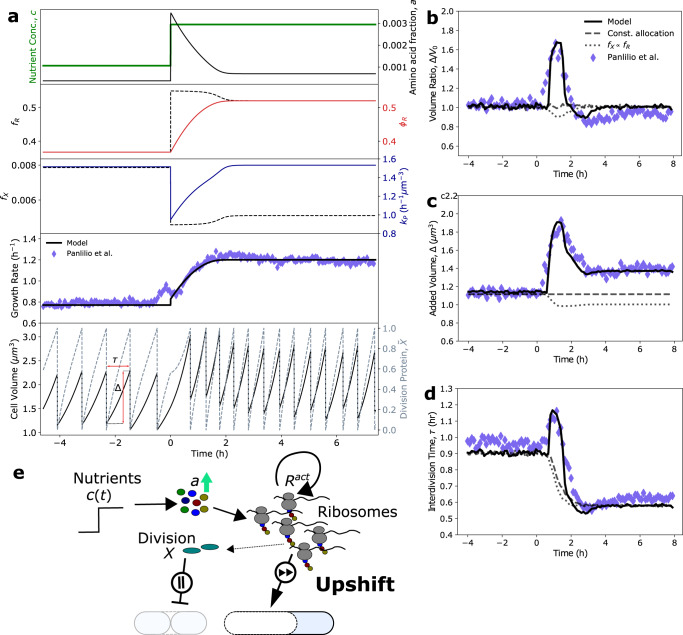
Cell size and division dynamics during nutrient upshift. **a** Simulation dynamics of average amino acid mass fraction, proteome composition, allocation fraction, and division protein production for the P5ter promoter strain of *E. coli* K-12 BW25113 cells undergoing nutrient upshift from M9 minimal medium with 0.4% glucose to M9 with 0.4% glucose and 0.5% casamino acids^5^. ${\kappa }_{t}^{0}$ and *μ*_*X*_ were obtained by fitting growth rate and size dynamics to experimental data, while the remaining parameters were calculated from steady-state proteomics and size data (see “Methods”). All parameters are provided in Table 1. Bottom: Single-cell volume trajectories were simulated using the model by implementing division rules appropriate for *E. coli*. **b**–**d** Generation-averaged dynamics of cell volume ratio (**b**), added volume (**c**), and interdivision time (**d**) from 400 single-cell volume trajectories agree well with experimental data. **e** Resource allocation strategy during upshift. In response to amino acid influx, cells transiently prioritize (fast forward) ribosome production over division protein production, resulting in an acceleration of biomass accumulation and a delay in division (pause).

Recent experimental results show that in response to nutrient upshift, bacteria transiently delay division before increasing to a faster division rate in nutrient-rich media^5^. This behavior is seen clearly in the overshoot in the average interdivision time (*τ*) and added volume (Δ) (Fig. 3c, d). Previous work has suggested that division control is co-regulated both by chromosome replication and a chromosome-independent accumulation process, such that the slowest process sets division timing in unperturbed cells^40^. Importantly, replication-initiation models which can accurately capture steady-state cell size control behavior are not able to capture these overshoot dynamics^5^, indicating that a chromosome-independent regulatory mechanism is likely responsible for size control in dynamic nutrient environments. We hypothesized that this division delay upon nutrient upshift results from cellular prioritization of ribosome production over production of division and metabolic proteins. Using our four-component proteome sector model, we simulated single-cell growth and size dynamics in response to nutrient upshift, and were able to quantitatively capture the experimental results (Fig. 3a), as well as predict the dynamics of flux allocation and proteome composition. Importantly, our model was also able to capture growth rate dynamics during both upshift and downshift in many other experimental conditions examined recently by Erickson et al.^18^ (Supplementary Fig. 3).

We simulated stochastic single-cell volume trajectories by introducing both growth rate and division noise during the cell cycle, in which only one daughter cell was tracked after each division event (Fig. 3a, bottom panel; see “Methods”). These simulations quantitatively captured the average added volume (Δ), volume ratio (Δ/*V*_0_), and the interdivision time (*τ*) dynamics seen experimentally (Fig. 3b–d). In particular, they reproduce the overshoot in added volume and interdivision time following the nutrient upshift. As hypothesized, our model predicts that in response to increased nutrient availability, bacteria transiently divert resources away from division and metabolic protein production and instead prioritize ribosome production (Fig. 3e). This regulatory behavior occurs because an increase in nutrient availability transiently causes a mismatch in the translational and metabolic fluxes, yielding a significant increase in the size of the amino acid pool, *a*. In response to the increase in *a*, the cell allocates translational flux to ribosome production at the expense of division and metabolic protein production (Fig. 1c, d). This is seen in the temporary drop in division protein production rate, *k*_*P*_, and overshoot in ribosomal flux allocation, *f*_*R*_, during the growth rate adaptation time period, after which both *k*_*P*_ and *f*_*R*_ relax to their new steady-state values (Fig. 3a). Consequently, during this transitional period, bacteria delay division and add significantly more biomass than their birth size (Fig. 3b). Importantly, resource allocation strategies in which division protein allocation is constant or co-regulated with ribosomal protein expression could not explain the experimentally-observed overshoot dynamics or steady-state size relationships (Fig. 3b–d, simulation details for alternative allocation strategies in Supplementary Note 5), clearly showing the necessity of *ϕ*_*X*_ to be co-regulated with *ϕ*_*P*_. We also found that inclusion of a nonzero degradation rate was necessary to capture the observed amplitude of the overshoots (Supplementary Fig. 2), consistent with observations that certain key division proteins like FtsZ are actively degraded by the cell^25,26,41^.

### Growth-rate and cell size recovery time increases with nutrient pulse duration

**Fig. 4:**
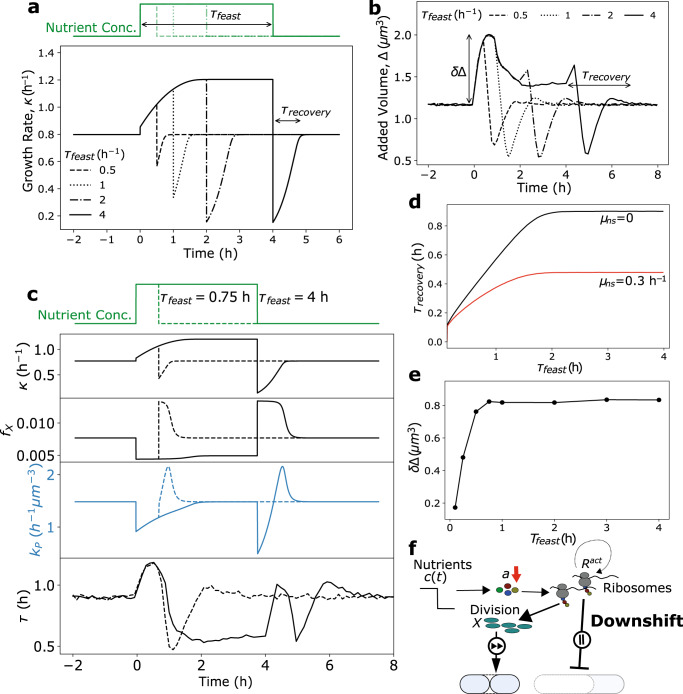
Proteome reallocation and cell size regulation in pulsatile nutrient environment. **a** Average single-cell growth rate simulations of bacteria experiencing a nutrient-rich pulse of variable duration. For each trajectory with pulse-length *τ*_feast_, the time required following downshift for the growth rate to return to within 99% of the pre-shift level was measured, given by *τ*_recovery_. **b** Average dynamics of added volume, Δ, for 400 single-cell trajectories experiencing a nutrient-rich pulse as shown in **a**. The time required to stabilize at the initial added volume after pulse cessation is again given by *τ*_recovery_. **c** Example simulation dynamics where *τ*_feast_ = 0.75 h, and *τ*_feast_ = 4 h. In both cases, the top four panels are deterministic simulations of average intracellular dynamics, whereas the bottom panel is the average dynamics of 400 single-cell stochastic simulations. **d** Quantification of the relationship of *τ*_feast_ and *τ*_recovery_ from the simulations in **a** for two different degradation rates. **e** Quantification of the relationship of *τ*_feast_ and *τ*_recovery_ from the simulations in **b**. See Table 1 for a list of model parameters. **f** Resource allocation strategy during downshift. In response to amino acid depletion, cells transiently prioritize (fast forward) division protein production, resulting in accelerated division and delayed biomass accumulation (pause).

To predict bacterial growth rate and cell size control in more complex time-varying environments, we simulated single-cell trajectories experiencing a pulse of nutrient-rich media. For each trajectory with pulse-length *τ*_feast_, we measured the time required (*τ*_recovery_) for both the growth rate and cell volume added per generation to return to the pre-shift level following downshift (Fig. 4a, b). Interestingly, in both cases *τ*_recovery_ increased with increasing *τ*_feast_ until saturating at a constant value (Fig. 4d), showing that bacteria transiently retain memory of the previous nutrient environment across generations, allowing for quicker recovery to optimal steady-state growth when experiencing short timescale perturbations in nutrient quality. As cellular growth rate is determined by ribosome abundance (Eq. (1)), we hypothesized that this phenotypic memory is conferred by the slow dynamics of proteome reallocation and thus ribosome accumulation, which occur on a significantly slower timescale than translational flux reallocation due to the half-life of proteins far exceeding that of mRNA^42^. As such, even though translation proceeds largely at the same rate as transcription in bacteria^43^, the stability of previously translated genes allows for transmission of previous metabolic information across time by increasing the time required to reshape proteome composition^24^.

In agreement with our hypothesis, we found that the time period over which cells maintain a memory of the previous state is equal to the time required to reshape the proteome to become optimal in the new environment (Figs. 3a and 4d). In addition, the recovery time and the duration of the phenotypic memory could be reduced by increasing the nonspecific protein turnover rate (Fig. 4d). These results show that the delay between translational flux reallocation and reorganization of the proteome incurs a short term fitness cost by slowing adaptation, but confers a fitness advantage in fluctuating conditions as it allows cells to quickly return to optimal growth in the previous condition if the nutrient perturbation is short-lived. This phenotypic memory is also predicted to occur during starvation (Supplementary Fig. 4), and is seen experimentally^44,45^. Specifically, cells which experience a longer starvation period take longer to recover their pre-starvation growth rate. In this regime, an analytical expression relating the starvation time to the recovery time can be obtained (Supplementary Note 6).

As with *τ*_recovery_, the overshoot in added volume, *δ*Δ, also increases with *τ*_feast_, but saturates at a constant value much quicker than *τ*_recovery_ (Fig. 4e). This is because the added volume is not determined solely by proteome allocation, but instead is given by the relative rates of biomass accumulation and division protein production (Δ ≈ *κ*/*k*_*P*_).

### Cell division is prioritized over biomass accumulation during nutrient downshift

Following cessation of the nutrient-rich pulse, our model makes the interesting prediction that division is prioritized over biomass accumulation during downshift (Fig. 4f), as allocation to division protein synthesis transiently becomes maximal at the expense of ribosome allocation (Fig. 1d). This behavior can be understood by recalling that *f*_*X*_ and *f*_*P*_ are co-regulated, and that an increase in *f*_*X*_ necessarily requires a decrease in *f*_*R*_ (Eq. (3)). As a result, there is a temporary increase in division rate (undershoot in *τ*) caused by an overshoot in *k*_*P*_ (Fig. 4c), while biomass accumulation temporarily slows (undershoot in *κ*, Fig. 4c), leading to a rapid reduction in cell size (undershoot in Δ, Fig. 4b). This prioritization of division protein synthesis is a surprising prediction given that following downshift, cells are experiencing harsher environmental conditions. We propose explanations for this behavior in the Discussion section.

Remarkably, our model predicts distinct pulse length-dependent recovery behavior in interdivision time following cessation of the nutrient-rich pulse. This can be seen clearly by comparing the simulation dynamics of bacteria experiencing nutrient-rich pulses of 0.75 and 4 hrs (bottom panel, Fig. 4c). Specifically, cells experiencing longer pulse lengths exhibit a non-monotonic recovery of interdivision time, *τ*, which is not observed at shorter pulse durations. This behavior can be understood by considering the impacts of both the overall translational flux (*J*_*t*_ = *κ*) and the division protein allocation fraction (*f*_*X*_) on division protein synthesis rate, given by *k*_*P*_ = *γJ*_*t*_*f*_*X*_. Under both conditions, *f*_*X*_ behaves similarly immediately following downshift, namely that allocation to division protein production transiently increases before relaxing to its steady-state value (third panel from top, Fig. 4c). As *k*_*P*_ is proportional to *f*_*X*_, at short pulse lengths the increase in *f*_*X*_ causes an overshoot in *k*_*P*_ following downshift (fourth panel from top, Fig. 4c). Importantly, there is a temporary undershoot in growth rate following downshift under both conditions, however the magnitude of this growth rate undershoot is significantly larger at longer pulse lengths (second panel from top, Fig. 4c) due to a greater mismatch in metabolic and translational fluxes. As *k*_*P*_ is also proportional to *κ*, at longer pulse lengths the initial drop in *k*_*P*_ is due to a temporary halt of translation. This is followed by a translation flux ramp-up in which division is prioritized, resulting in a temporary overshoot in *k*_*P*_, and an overall non-monotonic recovery behavior in *τ*. Importantly, when the quality of the nutrient-rich media is reduced but the pulse length remains long, there is a reduced growth rate undershoot following pulse cessation, and the non-monotonic recovery in *τ* is not seen (Supplementary Fig. 5). Thus, this pulse length-dependent division control is a direct consequence of the dependence of *k*_*P*_ on both *f*_*X*_ and *κ*.

### Cell size-dependent protein synthesis regulates recovery from stationary phase under pulsed nutrient supply

When the environmental nutrient supply has been exhausted, bacteria halt growth and enter stationary phase. Bacterial division control and size homeostasis behavior is markedly different in stationary phase compared to exponential phase, and a robust mechanistic model which captures size control dynamics in both phases of growth is still lacking. As such, we were interested if our model would successfully predict cell size and division control upon exit from stationary phase. Under such conditions, the effects of protein turnover on cell physiology become crucial^46^. From Eq. (1), we see that although bacterial growth vanishes in stationary phase (*κ* = 0), protein production does not cease completely, but is balanced by the degradation rate such that the translational flux is given by ${J}_{t}={\mu }_{{{{{{{{\rm{ns}}}}}}}}}={\kappa }_{t}({\phi }_{R}-{\phi }_{R}^{\min })$. This implies that a small fraction of ribosomes remain active and that amino acid supply comes solely from protein turnover. Importantly, division protein production scales with cell volume and persists in stationary phase, with *k*_*P*_ = *γf*_*X*_*μ*_ns_. As a result, the concentration of accumulated division proteins, *c*_*X*_, at steady-state in stationary phase is set by the relative rates of protein production and degradation, namely *c*_*X*_ = *γf*_*X*_*μ*_ns_*X*_0_/*μ*_*X*_, predicting that cells maintain a constant concentration of division proteins in stationary phase, regardless of cell size. Because division is dependent on the total number of accumulated division proteins and not its concentration, we therefore expect larger cells to divide faster upon nutrient exposure.

**Fig. 5:**
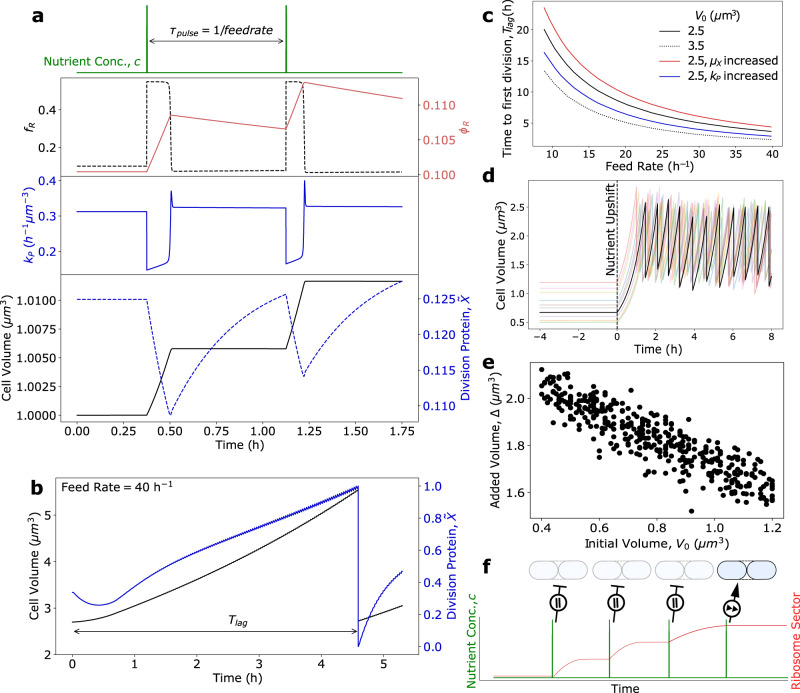
Cell size and division control during exit from stationary phase. **a** Single-cell dynamics of ribosome allocation fraction and mass fraction, division protein production, cell volume, and accumulated X protein abundance for *E. coli* experiencing pulses of nutrients with delay *τ*_pulse_ starting from stationary phase. **b** Cell volume and accumulated division protein abundance dynamics for a feedrate of 40 h^−1^. **c** Using the simulation setup shown in **a**, the time from pulsing onset until the first division event, *T*_lag_ (example trajectory shown in **b**), was measured as a function of pulse frequency (feedrate) for several initial volumes, degradation rates, and division protein production rates. For increased degradation, *μ*_*X*_ = 3 h^−1^. For increased *k*_*P*_, *γα* = 5.175μm^−3^ and *γβ* = 1.265μm^−3^. **d** Example single-cell trajectories of cells with randomized initial volumes exiting stationary phase via a single nutrient shift (dotted line). **e** Negative correlation (correlation coefficient −0.90) between initial cell volume (correlated to birth volume) and added volume for 400 simulations shows that *E. coli* exhibit sizer-like behavior when exiting stationary phase, in agreement with experimental observations^47^. See Table 1 for a list of model parameters. **f** In response to nutrient influx, cells temporarily decrease *k*_*P*_ to produce ribosomes. When a sufficient number of pulses have occurred such that *ϕ*_*R*_ abundance is no longer growth rate limiting, resources are reallocated to division.

To examine if our model is able to capture division control during transitions between different growth regimes, we simulated size control dynamics during stationary phase rescue. Given that our model predicts prioritization of biomass accumulation over division during upshift, we simulated rescue through repeated short-exposure nutrient pulses to examine the effects of repeated upshifts on size control and resource allocation. Specifically, cells starting at steady-state where *κ* = 0 and *c* = 0 experienced nutrient pulses of constant duration with a variable separation time, *τ*_pulse_ (Fig. 5a). As an increase in available nutrients results in an increase in the intracellular amino acid mass fraction^25^ (Supplementary Fig. 6), our model predicts that bacteria transiently prioritize ribosome production over division immediately following pulse exposure, similar to nutrient upshift behavior predicted in exponential phase (Figs. 5a and 3 and Supplementary Fig. 6). Consequently, immediately following nutrient influx, *k*_*P*_ drops and the degradation rate dominates, resulting in a sharp decrease in the division protein number, *X*. Importantly, in the time between pulses, *X* increases significantly due to an increase in division protein production caused by an increase in cell volume. This stands in contrast to a previous model for division control in stationary phase by Sekar et al.^25^, which assumed that bacteria immediately allocate resources to division during nutrient upshift, causing the division protein production rate to transiently increase before falling to some basal value if the pulse rate is of insufficient frequency. Despite the stark differences in molecular details between these models, we find that the time from pulse onset to first division, *T*_lag_ (Fig. 5b), monotonically decreases with increasing feedrate (decreasing *τ*_pulse_, Fig. 5c), which is observed experimentally^25^. This behavior occurs because although bacteria initially prioritize ribosome production over division when exiting stationary phase, once the ribosome bottleneck is relieved, cells then upregulate division machinery (Fig. 5f). As a faster feedrate relieves this bottleneck quicker, a faster feedrate results in a shorter lag time until division. Also consistent with experimental results^25^, we find that increasing the division protein degradation rate (*μ*_*x*_) increases *T*_lag_, while increasing the protein production rate (*k*_*P*_) decreases *T*_lag_ (Fig. 5c), highlighting the importance of the degradation and volume-specific protein synthesis rates in controlling division timing.

As cells in stationary phase maintain a constant concentration of division proteins regardless of size, our model predicts that *T*_lag_ is dependent on initial volume in stationary phase, *V*_0_, such that larger cells divide faster (Fig. 5c). Importantly, this dependence of division timing on initial cell size is seen experimentally^47,48^, and is not captured by the model proposed by Sekar et al.^25^. To more specifically investigate size control mechanisms when exiting stationary phase, we simulated single-cell volume trajectories of bacteria exiting stationary phase via a single nutrient upshift (Fig. 5d; see “Methods”). Importantly, we found that the adder model for cell size control did not hold under this growth regime, but rather cells exhibited sizer-like behavior, which is characterized by the added volume being negatively correlated with birth volume (Fig. 5e). This behavior has been observed experimentally^47^, and again can be understood from our threshold accumulation model, now considering the limit when *μ*_*X*_ ≫ *κ*. In such environments, bacteria divide once reaching a set size given by *V*_*d*_ = *μ*_*X*_/*k*_*P*_.

## Discussion

We have developed a coarse-grained proteome sector model which quantitatively captures experimentally observed growth rate and size control dynamics in response to nutrient upshift in both exponential (Fig. 3) and stationary phases (Fig. 5). Although the model has been derived based on data from *E. coli*, other bacterial species exhibit similar behavior^49^. The theoretical framework of dynamic flux balance and proteome allocation theory generalizes to other microorganisms, and the threshold accumulation rules for division are applicable for all bacterial species which exhibit adder or sizer-like size control. Using proteomics data in conjunction with cell size data for a particular organism, the various proteomic and kinetic parameters can be elucidated in our model, allowing for quantitative prediction of cell size and growth control in time-varying environments.

**Fig. 6:**
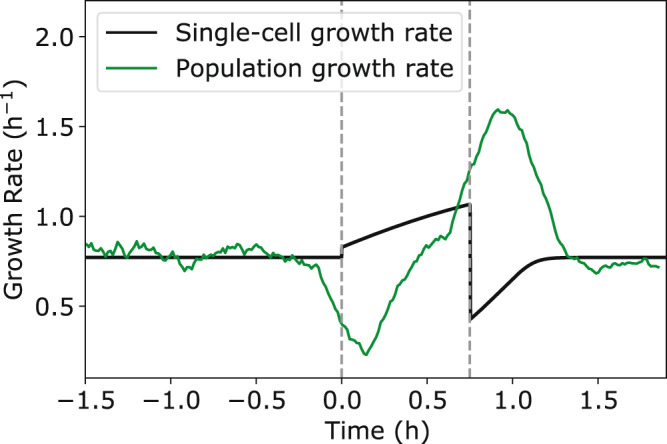
Cell proliferation dynamics during nutrient upshift. Comparison of single-cell growth rate (black) and population growth rate (green) dynamics in response to a nutrient-rich pulse, where the population growth rate is given by the relative rate of increase in the total number of cells over time. Dotted lines correspond to the start and end of the nutrient-rich period. Population growth simulations were initialized with 90 cells with randomized volumes. Following a division event, both resulting daughter cells were simulated. See “Methods” for simulation details and Table 1 for a list of model parameters.

Our model highlights an important resource allocation trade-off that cells must make between optimizing for biomass accumulation or division in dynamic nutrient environments. In response to nutrient upshift, we predict that bacteria prioritize ribosome production in both exponential and stationary phase, resulting in faster biomass accumulation but delayed division. At the single cell level, this results in a transient overshoot in both added volume and interdivision time. Interestingly, when simulating population-level growth dynamics (see Methods), we find that upshift causes a temporary reduction in population growth rate (Fig. 6). This raises the question, in response to increased nutrient availability, why do bacteria temporarily slow proliferation? One possible explanation is that by delaying division, cellular resources are freed up which can be reallocated to quickly alleviate the growth bottleneck caused by a lack of ribosomes. As a result, bacteria are optimized for biomass accumulation instead of population growth, which allows for individual cells to adapt quickly to new environments. A second explanation is that because bacteria can quickly inactivate ribosomes^23^ and recycle the amino acids through degradation, cells prioritize ribosome production as a method of energy storage when the environment is transiently nutrient-rich. Thus by producing ribosomes in response to nutrient upshift, bacteria simultaneously relieve the growth bottleneck caused by lack of ribosomes, while also quickly converting metabolites into proteins which can be reallocated in the future after nutrients have been exhausted. This strategy could allow for bacterial survival in harsher fluctuating environments, when nutrients are few and far between.

With our model able to capture nutrient upshift dynamics, we simulated bacterial growth rate and size control dynamics in response to pulsatile nutrient exposure to predict how resources are allocated in more complex time-varying environments. In such conditions, growth rate recovery time following downshift increased with pulse length (Fig. 4), showing that bacteria exhibit a transient memory of the previous metabolic state. This phenotypic memory arises from the slow dynamics of proteome reallocation, and although it incurs a short term fitness cost, this passive mechanism can confer a fitness advantage in fluctuating conditions, as it allows cells to quickly return to optimal growth in the previous condition if the nutrient perturbation is short-lived.

Our model also yielded surprising predictions for the size control dynamics following nutrient downshift (Fig. 4c). In particular, our model predicts that bacteria transiently prioritize production of division proteins over production of ribosomes, resulting in a temporary reduction in interdivision time and added volume. This result is striking, because it predicts that in response to the onset of harsher environmental conditions, bacteria transiently upregulate the production of costly division machinery instead of prioritizing energy storage. In addition, this prioritization of division results in a temporary overshoot in population growth rate (Fig. 6), meaning that the number of cells that must compete with each other for nutrients sharply increases in the new, less-favorable, environment.

Several potential explanations for this behavior warrant exploration in future experimental and theoretical studies. First, by transiently increasing division frequency while reducing biomass accumulation, bacteria rapidly decrease cell size and thus increase surface-to-volume ratio^3,50^. As a higher surface-to-volume ratio results in greater nutrient influx^51,52^, decreasing cell size may confer an important fitness advantage despite the metabolic cost associated with upregulating division protein production. In addition, smaller cells are better able to preserve membrane integrity and survive starvation compared to larger cells^53,54^, suggesting that the reduction in cell size in response to nutrient downshift is part of a cell level response to prepare for potential starvation. Lastly, bacteria may employ this increased rate of division as a population bet-hedging strategy which facilitates adaptation to fluctuating environments. Previous work has shown that partitioning of cellular contents at division is a major determinant of phenotypic heterogeneity^55^. Thus, by transiently increasing the number of division events, a bacterial population will temporarily exhibit a broader range of phenotypes. Phenotypic heterogeneity increases in adverse environments in both prokaryotic and eukaryotic populations, and previous work has shown that heterogeneity promotes adaptation to time-varying stress by facilitating development of resistance-conferring mutations, alleviating the fitness cost of constitutive expression of unnecessary proteins, and/or allowing for exploration of new phenotypes better suited for the harsher environment^56–60^. These results suggest that bacterial cells utilize division control to increase population heterogeneity in response to harsh environmental perturbations, thus facilitating adaptation to new environments and conferring increased population fitness in time-varying environments.

## Methods

### Model derivation

To model cellular growth rate and division control, we developed a four-component coarse-grained proteome sector model consisting of housekeeping proteins (*Q*), ribosomal proteins (*R*), metabolic proteins (*P*), and division proteins (*X*). Bacterial cells grow exponentially in size during the cell cycle. Assuming constant protein density, the growth rate *κ* of a single cell can be defined in terms of cell volume, *V*, or equivalently in terms of total protein mass, *M*, yielding

8 $$\kappa =\frac{1}{M}\frac{{{{{{{{\rm{d}}}}}}}}M}{{{{{{{{\rm{d}}}}}}}}t}=\frac{1}{V}\frac{{{{{{{{\rm{d}}}}}}}}V}{{{{{{{{\rm{d}}}}}}}}t},$$

The rate of change of protein mass is proportional to the mass of actively translating ribosomes. In addition, if we assume that protein turnover is governed by a constant, nonspecific degradation rate *μ*_ns_, then the rate rate of change of protein mass is given by

9 $$\frac{{{{{{{{\rm{d}}}}}}}}M}{{{{{{{{\rm{d}}}}}}}}t}={\kappa }_{t}\left({M}_{R}-{M}_{R}^{{{{{{{{\rm{in}}}}}}}}}\right)-{\mu }_{{{{{{{{\rm{ns}}}}}}}}}M,$$

where *κ*_*t*_ is the translational efficiency of the cell, *M*_*R*_ is the total mass of ribosomes, and ${M}_{R}^{{{{{{{{\rm{in}}}}}}}}}$ is the mass of inactive ribosomes. Using Eqs. (8) and (9), the growth rate can then be defined as $\kappa ={\kappa }_{t}({\phi }_{R}-{\phi }_{R}^{\min })-{\mu }_{{{{{{{{\rm{ns}}}}}}}}}$ where *ϕ*_*R*_ = *M*_*R*_/*M* is the ribosome mass fraction and ${\phi }_{R}^{\min }={M}_{R}^{{{{{{{{\rm{in}}}}}}}}}/M$ is the mass fraction of inactive ribosomes, thus recovering Eq. (1). To obtain the dynamics of *ϕ*_*R*_, we note that $\frac{{{{{{{{\rm{d}}}}}}}}{M}_{R}}{{{{{{{{\rm{d}}}}}}}}t}={\kappa }_{t}{f}_{R}({M}_{R}-{M}_{R}^{{{{{{{{\rm{in}}}}}}}}})-{\mu }_{{{{{{{{\rm{ns}}}}}}}}}{M}_{R}$, where *f*_*R*_ is the fraction of total cellular protein synthesis flux devoted to ribosomes. It follows that the time dynamics of *ϕ*_*R*_ are then

10 $$\begin{array}{r}\frac{{{{{{{{\rm{d}}}}}}}}{\phi }_{R}}{{{{{{{{\rm{d}}}}}}}}t}={\kappa }_{t}(a)\left({\phi }_{R}-{\phi }_{R}^{\min }\right)({f}_{R}(a)-{\phi }_{R}).\end{array}$$

Both *κ*_*t*_ and *f*_*R*_ depend on the amino acid concentration in the cell, which in turn depends on the nutrient availability. We note that the inclusion of a nonspecific degradation term does not impact the dynamics of *ϕ*_*R*_, as all cellular proteins are assumed to be degraded at the same rate. To connect cellular growth rate to amino acid mass fraction (*a*) and nutrient availability we use the following condition for flux balance^16^: $\frac{{{{{{{{\rm{d}}}}}}}}a}{{{{{{{{\rm{d}}}}}}}}t}={\kappa }_{n}(a){\phi }_{P}-\kappa$, where *κ*_*n*_ is the nutritional efficiency of the cell and *ϕ*_*P*_ is the mass fraction of P-sector protein that are responsible for transporting nutrients into the cell. Using the constraint ${\phi }_{R}+{\phi }_{P}+{\phi }_{X}=1-{\phi }_{Q}={\phi }^{\max }$, along with our definition of growth rate from Eq. (1), the amino acid mass fraction can be rewritten as

11 $$\frac{{{{{{{{\rm{d}}}}}}}}a}{{{{{{{{\rm{d}}}}}}}}t}={\kappa }_{n}(a)({\phi }^{\max }-{\phi }_{R}-{\phi }_{X})-{\kappa }_{t}(a)\left({\phi }_{R}-{\phi }_{R}^{\min }\right)+{\mu }_{{{{{{{{\rm{ns}}}}}}}}},$$

thus recovering Eq. (4). We note that amino acid supply is now given by a combination of nutrient import and amino acid recycling due to protein turnover. To make explicit the dependency of the efficiencies, *κ*_*n*_ and *κ*_*t*_, on *a*, we define two regulatory functions, *f*(*a*) and *g*(*a*), as given by ref. ^28^. Specifically, we assume ${\kappa }_{n}={\kappa }_{n}^{0}(c)f(a)$ and ${\kappa }_{t}={\kappa }_{t}^{0}g(a)$, where ${\kappa }_{t}^{0}$ is a constant, and ${\kappa }_{n}^{0}$ is a function of the extracellular nutrient concentration *c*. The regulatory functions are then:

12 $$f(a)=\frac{1}{1+{(a/{a}_{n})}^{2}},$$

13 $$g(a)=\frac{{(a/{a}_{t})}^{2}}{1+{(a/{a}_{t})}^{2}},$$

where translation becomes significantly attenuated for amino acid concentrations below *a*_*t*_, and the amino acid supply flux becomes significantly attenuated by feedback inhibition for *a* above *a*_*n*_.

Similar to the dynamics of *ϕ*_*R*_, the dynamics of *ϕ*_*X*_ can be obtained starting from the dynamics of the mass of protein X, yielding

14 $$\frac{{{{{{{{\rm{d}}}}}}}}{\phi }_{X}}{{{{{{{{\rm{d}}}}}}}}t}={\kappa }_{t}\left({\phi }_{R}-{\phi }_{R}^{\min }\right)({f}_{X}-{\phi }_{X})-({\mu }_{X}-{\mu }_{{{{{{{{\rm{ns}}}}}}}}}){\phi }_{X},$$

where *f*_*X*_ is the fraction of total synthesis capacity of a cell devoted to making cell division proteins, and *μ*_*X*_ is the degradation rate of protein X. This degradation rate is specific to protein X and can be different than the nonspecific degradation rate *μ*_ns_. When *μ*_*X*_ ≈ *μ*_ns_, the last term on the right hand side of Eq. (14) is negligible and the equation takes the same form as Eq. (10), allowing the dynamics of each sector to be written in vector form, as presented in the main text in Eq. (2). To derive the time evolution of the accumulated amount of protein X per cell, Eq. (14) can be rewritten in terms of the concentration of *X*, *c*_*X*_ = *ϕ*_*X*_*ρ*_*c*_/*m*_*X*_, where *ρ*_*c*_ is the protein mass density of the cell and *m*_*X*_ is the mass of molecule X. We then get

15 $$\frac{{{{{{{{\rm{d}}}}}}}}{c}_{X}}{{{{{{{{\rm{d}}}}}}}}t}=\frac{{f}_{X}{\rho }_{c}}{{m}_{X}}(\kappa +{\mu }_{{{{{{{{\rm{ns}}}}}}}}})-{c}_{X}(\kappa +{\mu }_{X}).$$

If the number of accumulated division proteins per cell is *X* = *c*_*X*_*V* − X^*^, where X^*^ is the amount of division proteins at birth, then using Eqs. (8) and (15) we can obtain the time dynamics of *X*, where

16 $$\frac{{{{{{{{\rm{d}}}}}}}}X}{{{{{{{{\rm{d}}}}}}}}t}=\frac{{f}_{X}{\rho }_{c}}{{m}_{X}}(\kappa +{\mu }_{{{{{{{{\rm{ns}}}}}}}}})V-{\mu }_{X}X.$$

In this model, division is triggered at *t* = *τ* after the cell has accumulated a fixed number of X proteins such that *X*(*τ*) = *X*_0_. For simplicity, we normalize Eq. (16) by *X*_0_ to yield the time dynamics of the fraction of the accumulated number of division proteins required to trigger cell division, such that

17 $$\frac{{{{{{{{\rm{d}}}}}}}}X/{X}_{0}}{{{{{{{{\rm{d}}}}}}}}t}=\frac{{{{{{{{\rm{d}}}}}}}}\tilde{X}}{{{{{{{{\rm{d}}}}}}}}t}=\gamma {f}_{X}(\kappa +{\mu }_{{{{{{{{\rm{ns}}}}}}}}})V-{\mu }_{X}\tilde{X},$$

where *γ* = *ρ*_*c*_/*X*_0_*m*_*X*_ and here now cells divide when $\tilde{X}(\tau )=1$. This allows us to identify the division protein synthesis rate per unit volume, *k*_*P*_, given by *k*_*P*_ = *γf*_*X*_(*κ* + *μ*_ns_). As with the mass fractions, the allocation fractions are constrained such that ${f}_{R}+{f}_{P}+{f}_{X}=1-{f}_{Q}={\phi }^{\max }$, meaning that two regulatory functions must be defined in order to simulate the dynamics of growth rate and cell size control. To do so, we assume that allocation is dependent on the amino acid pool, and that the division protein sector (X) is partially co-regulated with the metabolic protein sector (P), such that *f*_*X*_(*a*) is given by a linear combination of two sub-sectors: ${f}_{X}^{\alpha }(a)$, which denotes the portion which is co-regulated with the metabolic sector, and *β*, which denotes the basal allocation fraction. If we define ${f}_{P}^{* }(a)={f}_{P}(a)+{f}_{X}^{\alpha }(a)$ as the proteome fraction which is co-regulated opposite of *f*_*R*_(*a*), then *f*_*X*_(*a*) can be expressed as a function of *f*_*R*_(*a*), such that

18 $${f}_{X}(a)={f}_{X}^{\alpha }(a)+\beta =\alpha {f}_{P}^{* }(a)+\beta =\alpha \left({\phi }_{R}^{\max }-{f}_{R}(a)\right)+\beta ,$$

where *α* is the fraction of ${f}_{P}^{* }$ which contains the co-regulated portion of division proteins and where ${\phi }_{R}^{\max }={\phi }^{\max }-\beta$. The fraction of total synthesis capacity devoted to production of ribosomes, *f*_*R*_(*a*), is given by

19 $${f}_{R}(a)=\frac{-{f}^{{\prime} }(a)g(a){\phi }_{R}^{\max }+f(a){g}^{{\prime} }(a){\phi }_{R}^{\min }}{-{f}^{{\prime} }(a)g(a)+f(a){g}^{{\prime} }(a)},$$

in which *f*_*R*_ is chosen to maximize amino acid flux at steady state (Supplementary Note 1 and Supplementary Fig. 7).

With *f*_*X*_ now defined, the division protein synthesis rate can now be rewritten in terms of *f*_*R*_, where

20 $${k}_{P}=\gamma \left(\alpha ({\phi }_{R}^{\max }-{f}_{R}(a))+\beta \right)(\kappa +{\mu }_{{{{{{{{\rm{ns}}}}}}}}}).$$

At steady state *k*_*P*_ can be rewritten solely as a function of growth rate, such that

21 $${k}_{P}(\kappa )=\gamma \left(\alpha \left(\Delta \phi -\frac{\kappa +{\mu }_{{{{{{{{\rm{ns}}}}}}}}}}{{\kappa }_{t}}\right)+\beta \right)(\kappa +{\mu }_{{{{{{{{\rm{ns}}}}}}}}}),$$

where $$\Delta \phi ={\phi }_{R}^{\max }-{\phi }_{R}^{\min }$$.

### Simulating stochastic single-cell volume trajectories

In our modeling of growth rate, amino acid, and proteome allocation dynamics, we simulated deterministic trajectories by numerically solving the coupled ODEs defined by Eqs. (2) and (4). These solutions predict the average single cell behavior. In order to investigate size control mechanisms, we must also include the growth rate and division noise observed in real biological systems. To this end, we also simulated single cell volume trajectories using a continuous-time stochastic hybrid system. In this setup, we introduce two sources of noise at the generational level. First, we consider growth rate noise. Although there is wide cell-to-cell variability in growth rate in the same nutrient environment, experimental data reveal that there is essentially no correlation between the growth rate of a mother and it’s daughter cells^61^. Thus at steady-state, growth rate noise can be implemented by representing *κ* as an independent random variable drawn from a normal distribution at cell birth^62^. We extend this framework into time-varying environments, and model growth rate noise in single cells by drawing an offset value, *δκ*, at the start of each new generation such that the growth rate for the *i*th cell is given by

22 $${\kappa }_{i}(t)=\langle \kappa (t)\rangle +\delta {\kappa }_{i},$$

where 〈*κ*(*t*)〉 is the population average growth rate at time *t*. Our analysis of single cell growth rate data^5^ shows that the distribution of growth rates remains approximately Gaussian throughout nutrient upshift (Supplementary Fig. 8a), and that the standard deviation of the distribution, *σ*_*κ*_ is a linear function of the growth rate, such that

23 $${\sigma }_{\kappa }=a\langle \kappa \rangle +b,$$

where *a* and *b* are parameters obtained from fitting *κ* vs. *σ*_*κ*_ data through the shift (Supplementary Fig. 8b, c). As a result, a single offset value, *δκ*, is drawn from a normal distribution with mean 0 and standard deviation *σ*_*κ*_ at the start of each cell cycle, and remains constant until cell division. Using this cell-specific growth rate, each volume trajectory can be computed using

24 $$\frac{{{{{{{{\rm{d}}}}}}}}{V}_{i}}{{{{{{{{\rm{d}}}}}}}}t}={\kappa }_{i}(t){V}_{i},$$

where this equation is coupled to Eq. (6) to determine cell division events. Cells do not divide exactly symmetrically, but instead exhibit partitioning error at division^63^. To implement this second source of noise into our simulation, the birth volume of the daughter cell, *V*_0_, is given by the previous generation’s final volume, *V*_*d*_, multiplied by a random variable *r*, which is drawn from a normal distribution with mean 0.5 and standard deviation 0.04^63^, such that

25 $${V}_{0}^{i+1}=r{V}_{d}^{i},$$

where *i* and *i* + 1 denote the mother and daughter generations, respectively.

Bacteria cease biomass accumulation in stationary phase. As such, introducing growth rate noise at the generational level when simulating exit from stationary phase in the manner described above is not feasible. Instead, to probe cell division control in our model, we introduced noise in $\tilde{{X}_{0}}$, the threshold value required to trigger cell division. Specifically, for each cell a unique value of $\tilde{{X}_{0}}$ was drawn from a normal distribution with mean 1 and standard deviation 0.05.

### Stochastic population-level simulations

Population-level simulations were carried out using the same procedure as the single cell stochastic volume simulations, except for at each division event, both daughter cells were tracked, leading to a growing number of cell trajectories over time. Symmetric division noise was implemented similar to the single cell simulations, with one value of *r* drawn at each division, yielding corresponding birth sizes for daughter cells 1 and 2 as

26 $${V}_{0,1}^{i+1}=r{V}_{d}^{i},$$

27 $${V}_{0,2}^{i+1}=(1-r){V}_{d}^{i}.$$

Growth rate noise was implemented the same way as in the single cell simulations, with an offset value drawn for each cell at birth.

Using the simulation method detailed above, we tracked the number of cells over time, *P*(*t*). To calculate the population growth rate, we consider population growth an exponential process and solve for the instantaneous growth rate, *κ*_pop_. We obtained the instantaneous growth rate by computing the discrete derivative of the natural logarithm of the total number of cells between each time point, specifically

28 $${\kappa }_{{{{{{{{\rm{pop}}}}}}}}}=\frac{\ln (P(t+\Delta t))-\ln (P(t))}{\Delta t\ln 2}.$$

### Obtaining parameter values from experimental data

Although proteome allocation strategies are conserved across bacterial strains, the exact abundance of each proteome sector is strain specific. Therefore, we expect parameter values to vary across different strains. Experimental data used to validate our dynamic model in Fig. 3 was from *E. coli* K-12 BW25113 with an inserted gfpmut2 gene controlled by a constitutive promoter located at the terminus of replication^5^. Unfortunately, this experiment did not test a wide range of nutrient conditions, so we had to use other data to test our steady state predictions. As a result, in Fig. 2 we used data from *E. coli* K-12 NCM3722 cells^12,26^, for which there is no data in dynamic nutrient environments. Although the parameter values change slightly between strains as expected, the general resource allocation strategies and size control behavior is robust to parameter choice (Fig. 3 and Supplementary Fig. 9).

In our dynamic model, there are two fitting parameters, ${\kappa }_{t}^{0}$ and *μ*_*X*_, which are obtained by fitting our model to the dynamic growth rate and size control data, respectively. All other parameters, namely *γα*, *γβ*, ${\kappa }_{n}^{0,{{{{{{{\rm{low}}}}}}}}}$, and ${\kappa }_{n}^{0,{{{{{{{\rm{high}}}}}}}}}$, can be inferred from experimental observables using our model equations evaluated at steady state. Specifically, for a given value of ${\kappa }_{t}^{0}$, there is a unique value of ${\kappa }_{n}^{0,{{{{{{{\rm{low}}}}}}}}({{{{{{{\rm{high}}}}}}}})}$ which yields the observed steady state growth rate, *κ*^low(high)^, for a given condition. This value can be obtained by solving Eqs. (1), (11), and (19). In the same way, for a given value of *μ*_*X*_, there exists a unique combination of *γα* and *γβ* which yields the observed steady state cell size at birth in both nutrient conditions. These values are obtained by solving the system of equations given by Supplementary Eq. (S.12) evaluated at both the nutrient rich and poor conditions.

### Data analysis

Single-cell size vs. growth rate data was acquired from the supplementary data of Taheri-Araghi et al.^12^ and Si et al.^26^. The growth rate, added volume, volume ratio, and interdivision time vs. time data was obtained from the supplementary data of Panlilio et al.^5^. Calculation of population-averaged or time-averaged growth rate and size control parameters from single-cell data was performed using custom codes written in python (version 3.8.3) or MATLAB (version 2021a). Numerical simulations were performed using custom codes written in python (version 3.8.3).

### Statistics and reproducibility

Simulation results were compared to either population-averaged or time-averaged experimental data, when appropriate. For stochastic model implementations, a sufficient number (>400) of trajectories were simulated such that the average behavior was invariant to additional stochastic realizations.

### Reporting summary

Further information on research design is available in the Nature Portfolio Reporting Summary linked to this article.

## Supplementary information

Supplementary Information

Reporting Summary

## Data Availability

Source data are available at https://github.com/BanerjeeLab/DynamicCellSize.

## Code availability

Custom computer codes that were used in this paper are available at https://github.com/BanerjeeLab/DynamicCellSize.
