# Supplementary material for: Dynamic proteome trade-offs regulate bacterial cell size and growth in fluctuating nutrient environments
Source: Commun Biol. 2023 May 5;6:486. doi: 10.1038/s42003-023-04865-4 (PMC10163005; doi:10.1038/s42003-023-04865-4)
Supplement: Supplementary file 1 — Supplementary Information [file 42003_2023_4865_MOESM1_ESM.pdf]

Supplementary Information

Dynamic proteome trade-offs regulate bacterial cell size and growth in fluctuating nutrient environments

Josiah C. Kratz and Shiladitya Banerjee

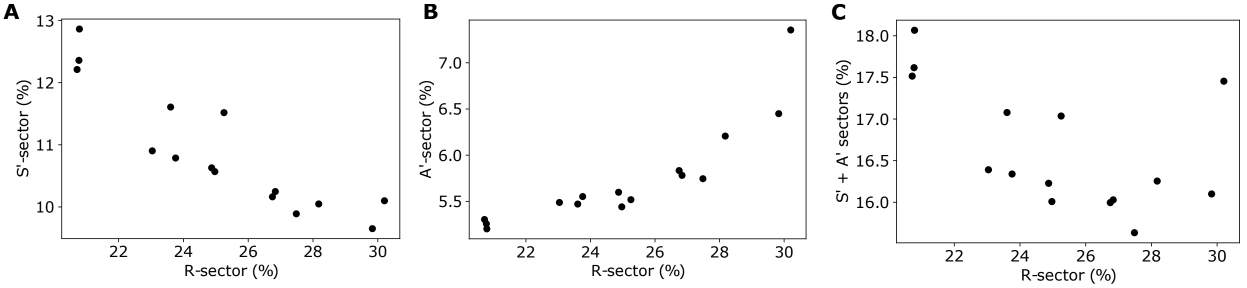

Supplementary Figure 1. **Abundance of division proteins across conditions**. The identities of the proteins responsible for determining division timing are not clearly known, making it difficult to determine the abundance of ϕ_X across conditions. However, there is experimental evidence of nutrient-dependent tradeoffs between ribosomal and division proteins from recent steady state proteomics data. Mori et al. [1] measured the proteome composition of *E. coli* under carbon, nitrogen, and translational limitation. Based on how the abundance of each protein changed under each limitation, the proteome was broadly classified into eight sectors, corresponding to whether each protein was upregulated or downregulated under each limitation. Two sectors were both highly enriched in proteins involved in cell cycle and division control, namely S' and A'. The abundance of these two sectors are shown as a function of the R-sector (ribosomal) in (**A**) and (**B**), as well as a combined plot of A'+S' in (**C**), which contains most of the cell division and cell cycle proteins. Under carbon limitation, the R-sector mass fraction decreases as both the S' and A'+S' sectors' mass fractions increase, in agreement with our model predictions. The fact that some division proteins increase with the R-sector (contained in the A'-sector), and thus increase with cell size, is not surprising given that certain division processes must scale with cell size (ex. a bigger septum is required for larger cells).

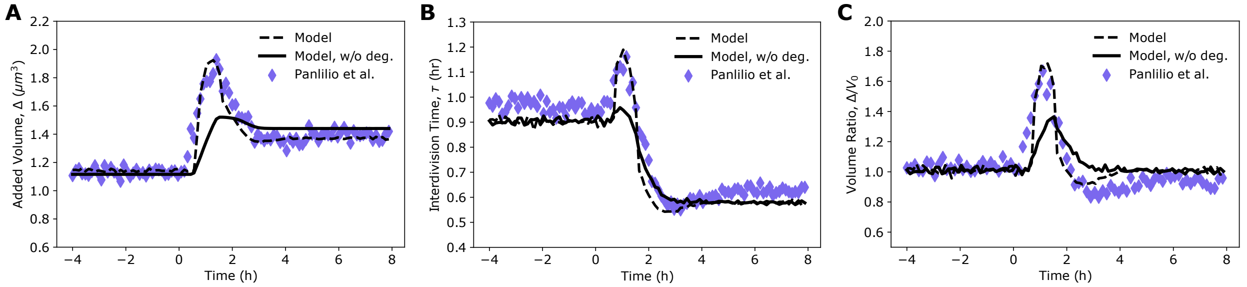

Supplementary Figure 2. **Inclusion of degradation rate, μ_X, is necessary to produce experimentally-observed size control overshoots**. Generation-averaged dynamics of added volume (A), interdivision time (B), and cell volume ratio (C) from 400 single-cell volume trajectories with (dotted) and without (solid) inclusion of the division protein degradation rate, μ_X. Increasing the degradation rate increases the amplitude of the size control overshoot.

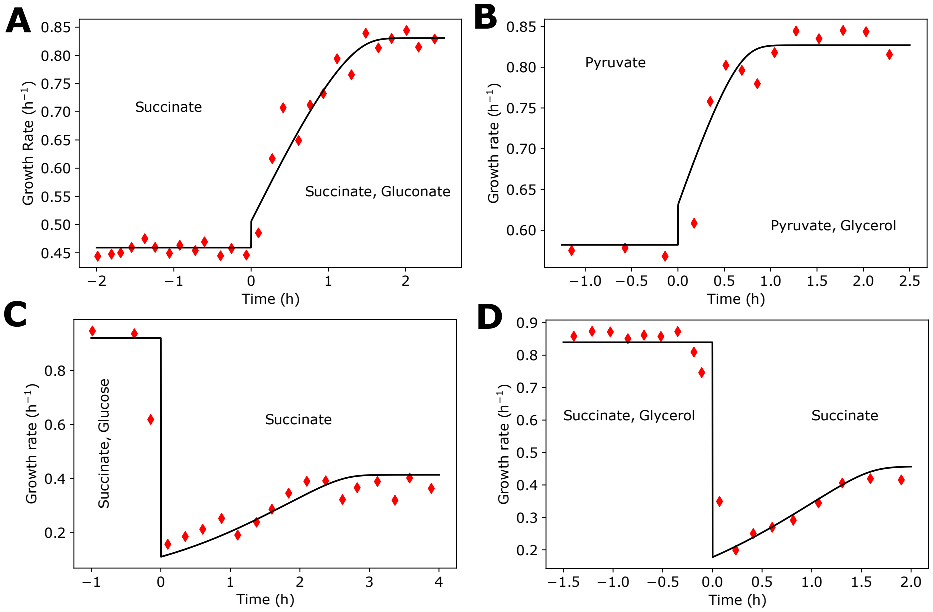

Supplementary Figure 3. **Validation of growth rate control model in multiple experimental conditions**. Model successfully predicts growth rate dynamics during nutrient upshift **A, B** and downshift **C, D** in multiple different experimental conditions from Ref. [2], specified in each figure. Specifically, these include upshift from 20 mM succinate to 20 mM succinate with 20mM gluconate, and upshift from 20 mM pyruvate to 20 mM pyruvate and 0.2% glycerol. Downshifts occurred from 20 mM succinate and 0.03% glucose to solely 20 mM succinate, and from 20 mM succinate with 1.11 mM glycerol to solely 20 mM succinate. κ_t^0 was obtained by fitting to growth rate dynamics, while $\kappa_{\mathrm{n,low}}^0$ and $\kappa_{\mathrm{n,high}}^0$ were calculated from the steady state growth rate in each nutrient condition (see Methods).

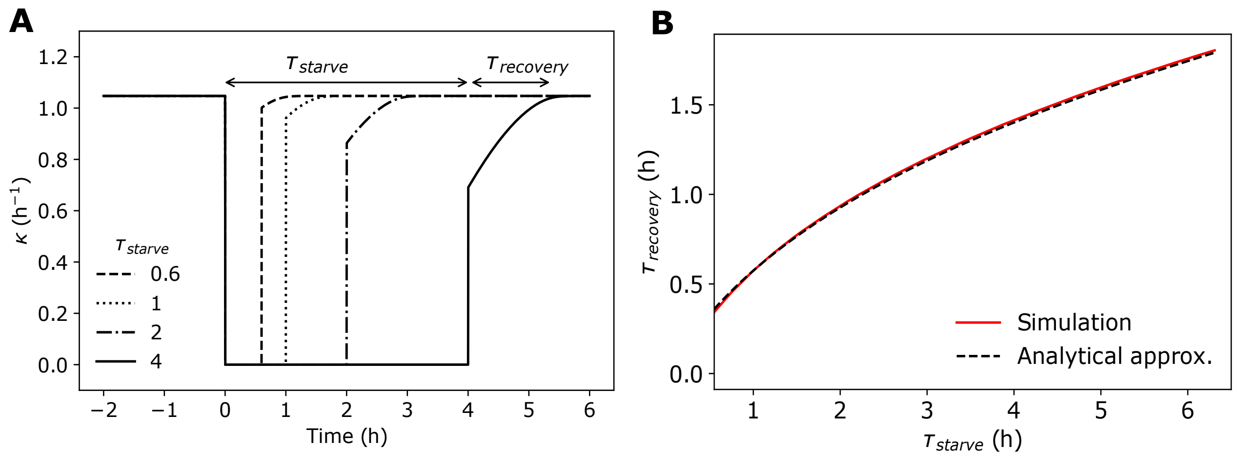

Supplementary Figure 4. **Recovery from stationary phase is dependent on starvation period.** **A** Average single-cell growth rate simulations of bacteria experiencing a nutrient-free pulse of duration τ_{starve}. For each trajectory with pulse-length τ_{starve}, the time required following downshift for the growth rate to return to within 99% of the pre-shift level was measured, given by τ_{recovery}. **B** Quantification of the relationship of τ_{starve} and τ_{recovery} from the simulations in **A**, along with the analytical approximation for comparison. Parameters are identical to those used in Figure 5 of the main text (given in Table 1), except $\kappa^0_{n,high} = 30\ \text{h}^{-1}$.

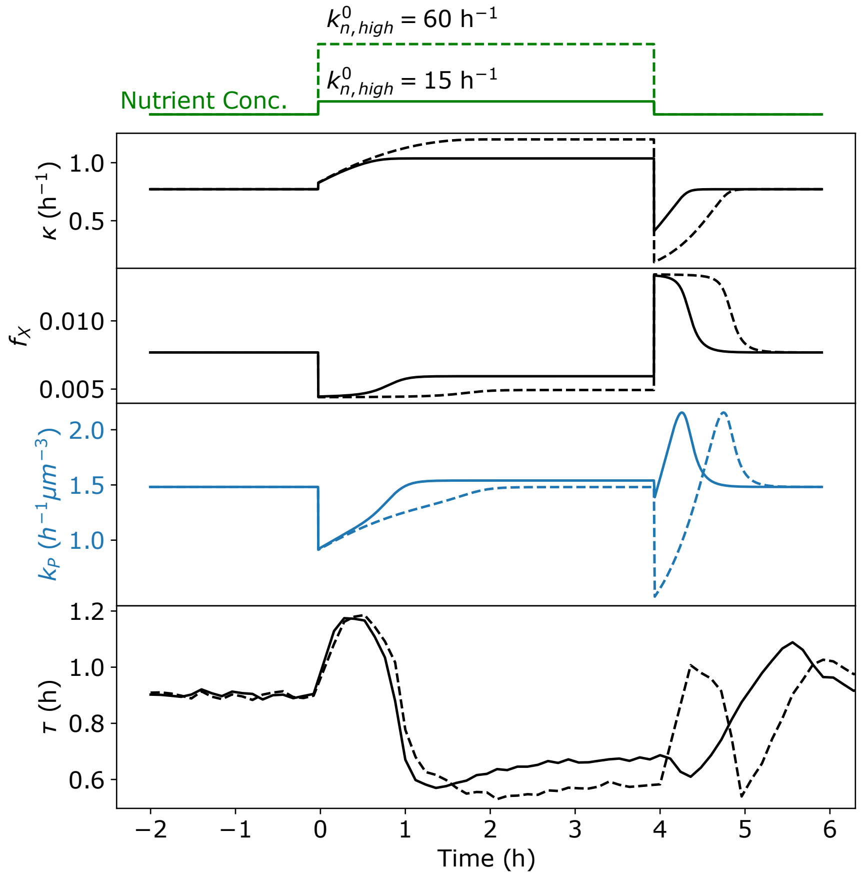

Supplementary Figure 5. **Interdivision time recovery behavior is pulse amplitude dependent.** Average single-cell growth rate simulations of bacteria experiencing a nutrient-rich pulse of duration $\tau_{\text{feast}} = 4$, with different nutrient concentrations during the nutrient-rich pulse. In both cases, the top four panels are deterministic simulations of average intracellular dynamics, whereas the bottom panel is the average dynamics of 400 single-cell stochastic simulations. Parameters are identical to those used in Figure 4 of the main text (given in Table 1), except for $k^0_{n,high}$ (given on top panel).

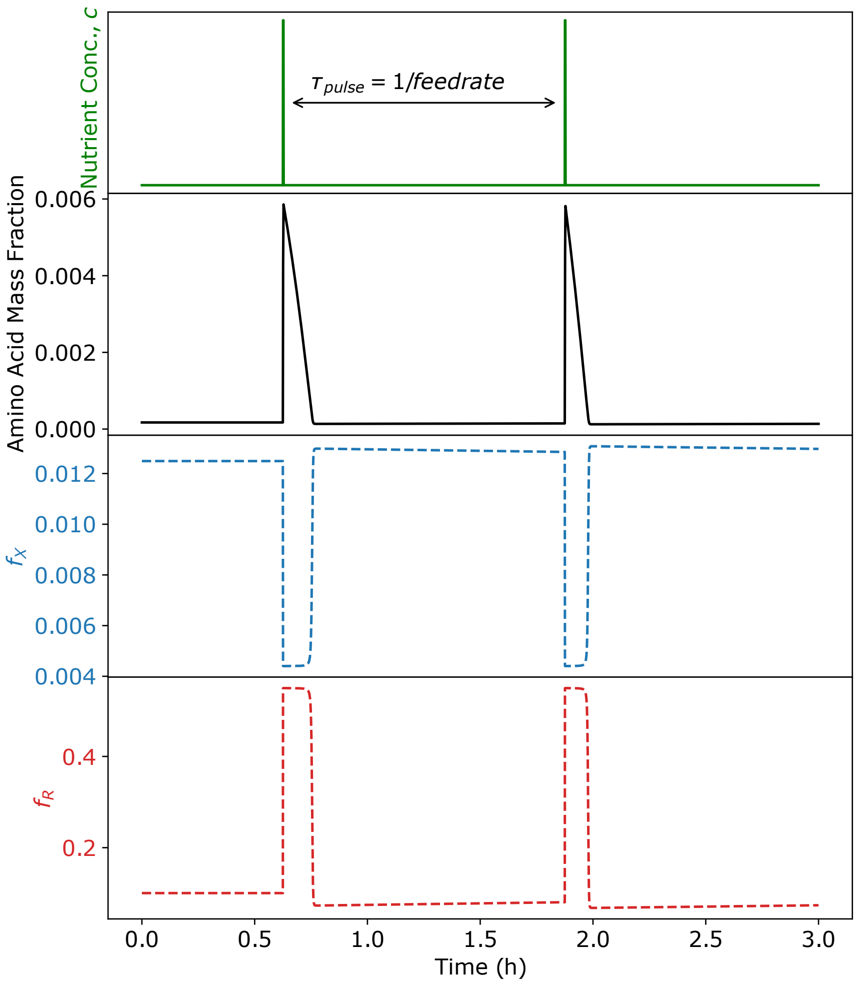

Supplementary Figure 6. **Dynamic resource allocation during exit from stationary phase**. Single-cell simulation dynamics of amino acid mass fraction and division protein allocation fraction for *E. coli* experiencing pulses of nutrients with delay τ_{pulse} starting from stationary phase. An increase in available nutrients results in an increase in the intracellular amino acid mass fraction. In response to the influx of resources, our model predicts that bacteria transiently prioritize ribosome production (f_R) over division (f_X) immediately following pulse exposure, similar to nutrient upshift behavior predicted in exponential phase. Parameters are identical to those used in Figure 5 of the main text (given in Table 1).

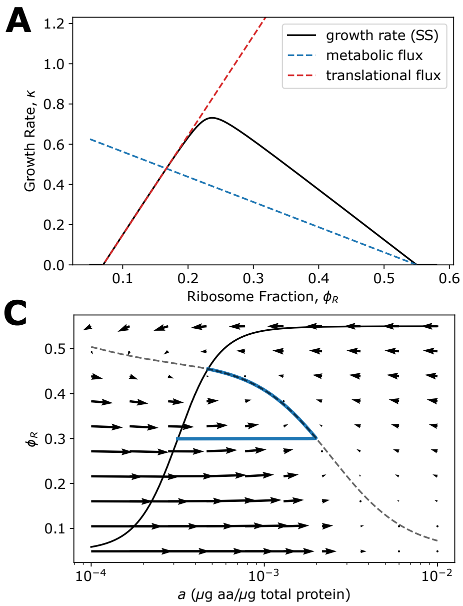

Supplementary Figure 7. **Flux balance by regulatory functions**. **A, B** Representative rate-balance plots of when translational and metabolic fluxes are balanced but growth is not maximal (A), and when fluxes are simulateneously balanced and maximized (B). f_R is defined such that for a given nutrient condition, f_R is equal to the ribosome fraction which both balances and maximizes flux. **C, D** Phase-plane analysis of model during nutrient upshift (C) and downshift (D). The model trajectory is shown in blue, showing behavior immediately after the nutrient shift occurs. The nullclines for ϕ_R and a are shown as solid and dashed curves, respectively.

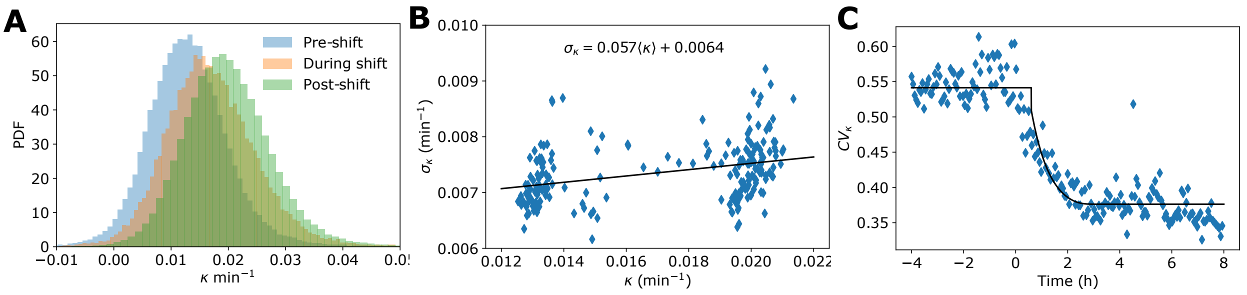

Supplementary Figure 8. **Simulating growth rate noise during nutrient upshift**. **A** Single cell growth rate distributions remains approximately Gaussian during nutrient upshift. Growth rate data from Ref. [3] are binned based on time of division into Pre-shift (-4-0 hrs), During shift (0-2.5 hrs), and Post-shift (2.5-8 hrs). **B** The standard deviation remained proportional to the average single cell growth rate during nutrient upshift, allowing time-binned growth rate (κ) vs standard deviation (σ_κ) data to be fit to a linear model to obtain parameters a and b, yielding $a = 0.057$ and $b = 0.0064$. **C** Using the relationship obtained in **B**, the time evolution of the coefficient of variation (CV_κ) during nutrient upshift (starting at $t = 0$) was accurately captured by simulating growth rate and standard deviation.

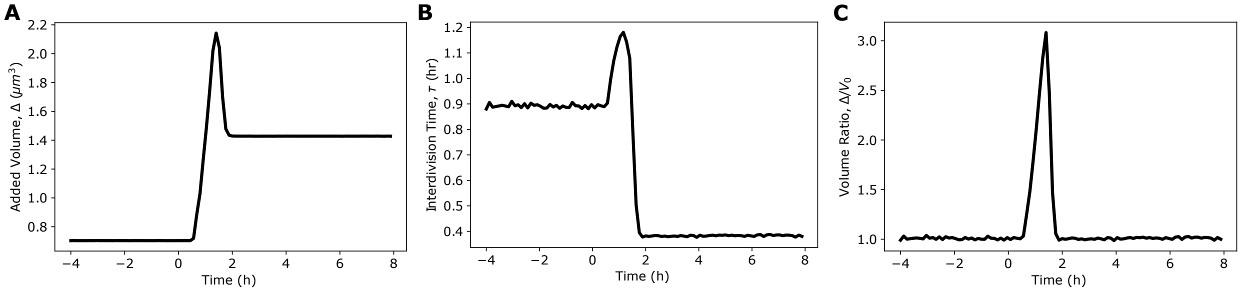

Supplementary Figure 9. **Size control behavior is robust to parameter choice**. Generation-averaged dynamics of added volume (A), interdivision time (B), and cell volume ratio (C) from 400 single-cell volume trajectories for parameters corresponding to data from Ref. [4]. Although proteome allocation strategies are conserved across bacterial strains, the exact abundance of each proteome sector is strain specific. Specifically here, $\kappa^0_{n,low} = 2.65$ h^{-1} and $\kappa^0_{n,high} = 21.2$ h^{-1}, corresponding to a transition between steady state growth rates of 0.78 h^{-1} and 1.82 h^{-1}, respectively. The remaining parameters are given in Table 1.

SUPPLEMENTARY NOTE 1: GROWTH RATE MAXIMIZATION TO OBTAIN RIBOSOMAL ALLOCATION FRACTION

During steady-state exponential growth, the rate of amino acid supply is balanced by the rate of amino acid consumption through protein synthesis to ensure that there is no net change in the amino acid concentration. Furthermore, the rate of protein synthesis equals the rate of bacterial growth, so the cell is faced with the dual objectives of balancing and maximizing the amino acid flux in order to maximize the growth rate (Supplementary Figure 7a,b). For a given translational efficiency $\kappa_t(a)$ and the nutritional efficiency $\kappa_n(a)$ (as determined by the growth medium), the organism must choose the ribosomal protein fraction $f_R(a)$ that balances the amino acid flux. This is implemented as follows [5]. At steady-state $\phi_R = f_R$ (Eq. (10)). Using the condition of steady-state in Eqs. (10) and (11) we get

$$f_R(a) = \frac{\kappa_n(a)\phi_R^{\max} + \kappa_t(a)\phi_R^{\min} + \mu_{\text{ns}}}{\kappa_n(a) + \kappa_t(a)} \,. \tag{S.1}$$

The steady-state growth rate is given by

$$\kappa(a) = \kappa_t(a)(f_R - \phi_R^{\min}) - \mu_{\text{ns}} \tag{S.2}$$

$$= \frac{\kappa_t(a)\Delta\phi - \mu_{\text{ns}}}{1 + \frac{\kappa_t(a)}{\kappa_n(a)}} \tag{S.3}$$

where $\Delta\phi = \phi_R^{\max} - \phi_R^{\min}$. Maximizing growth rate via $\kappa'(a) = 0$, we get the condition

$$\mu_{\text{ns}} = \frac{\kappa_n^0 g'(a) f(a)^2 \Delta\phi + \kappa_t^0 g(a)^2 f'(a)\Delta\phi}{g(a)f'(a) - g'(a)f(a)} \,. \tag{S.4}$$

Substituting the above expression in the equation for f_R we get the functional dependence of f_R on amino acid concentration.

$$f_R(a) = \frac{\kappa_n^0 f(a)\phi_R^{\max} + \kappa_t^0 g(a)\phi_R^{\min} + \mu_{\text{ns}}}{\kappa_n^0 f(a) + \kappa_t^0 g(a)} \tag{S.5}$$

$$= \frac{-f'(a)g(a)\phi_R^{\max} + f(a)g'(a)\phi_R^{\min}}{-f'(a)g(a) + f(a)g'(a)} \,. \tag{S.6}$$

SUPPLEMENTARY NOTE 2: DYNAMIC FLUX BALANCE AND MAXIMIZATION

Changes in available nutrient concentration, c, affect the metabolic flux through κ_n. As a result, in response to nutrient fluctuations, the cell must dynamically match the translational flux to the metabolic flux to prevent unsustainable build-up or depletion of the amino acid pool. The cell is able to alter both fluxes through three mechanisms which respond on two different timescales. The translational and nutritional efficiencies, κ_t and κ_n, depend only on the current cellular amino acid levels (Eqs. (12) and (13)), and

so can adjust quickly in response to changes in nutrient conditions. Following a nutrient shift, there is a sudden jump in a caused by a temporary mismatch in fluxes. The regulatory functions ensure that the fluxes quickly rebalance, eliminating any subsequent dramatic changes in a until the next change in c. The cell achieves further changes in flux by adjusting its ribosome mass fraction, ϕ_R, via adjustment to the fraction of ribosomes allocated to synthesize additional ribosomes, f_R. Importantly, altering ϕ_R via f_R is accomplished through protein synthesis and degradation, and thus occurs on a much slower timescale than changes to κ_t and κ_n. This behavior can be seen graphically using phase-plane analysis, in which the model trajectory first moves to the a nullcline by changing a without changing ϕ_R, at which point the fluxes have been rebalanced, before then moving to the fixed point by changing both a and ϕ_R (Supplementary Figure 7c,d).

SUPPLEMENTARY NOTE 3: ESTIMATING DIVISION PROTEIN ALLOCATION FRACTION FROM PROTEOMICS DATA

In order to obtain a value for allocation fraction f_X, the identity of the specific cell division proteins, collectively referred to as X proteins, must be known. Although multiple proteins may be involved in setting division timing, experimental evidence suggests that FtsZ is the main determinant of cell division control in *E. coli* [6, 7]. As such, in order to obtain approximate values for f_X we assume that X protein abundance is made up entirely of FtsZ proteins. Obtaining an estimate for f_X requires that the parameters α and β be known, given that $f_X = \alpha(\phi_R^{\max} - f_R) + \beta$. In order to estimate these parameters, Eq. (S.13) can be fit to experimental data, where there are three fitting parameters, $\gamma\alpha$, $\gamma\beta$, and κ_t ($\Delta\phi$ can be inferred from experimental data [8]), where $\gamma = \rho_c / X_0 m_X$. To find α and β explicitly, the value of γ must be known. To estimate its value, we assume that the division threshold, X_0, is determined entirely by FtsZ, such that $X_0 = M_X^0 / m_X$, where M_X^0 is the total mass of FtsZ at division, and m_X is the mass of a single FtsZ protein. As a result, we obtain the expression $\gamma = \rho_c / M_X^0$. Using proteomics data [9], we estimate that $M_X^0 \approx 6 * 10^{-4}$ pg. With the typical protein mass density of an *E. coli* cell given by $\rho_c \approx 0.15$ pg/μm^3 [10], the parameters α and β can be calculated using $\gamma \approx 250$ μm^{-3}. Interestingly, the range of proteome fractions obtained from our calculated size control parameters α and β are similar to the measured range of proteome fractions for FtsZ, namely $\sim 10^{-3}$ [1]. This further supports the notion that FtsZ is indeed responsible for division timing in *E. coli*.

SUPPLEMENTARY NOTE 4: GROWTH-RATE DEPENDENT CONTROL OF CELL SIZE

The coupled equations (8), (10), (11), and (17) in the Methods define the dynamics of the system. As X represents the accumulated number of division proteins, the amount of division molecules is reset to zero at cell birth (i.e. $\tilde{X}(0) = 0$). Thus, at steady state growth Eqs. (8) and (17) can be solved to obtain

$$\tilde{X}(t) = \frac{k_P V_0}{\mu_X + \kappa}(e^{\kappa t} - e^{-\mu_X t}) \, , \tag{S.7}$$

where V_0 is cell volume at birth. If cells divide symmetrically (i.e. $V_d = 2V_0$) at $t = \tau$ after accumulating the required number of X proteins such that $X(\tau) = X_0$ or equivalently $\tilde{X}(\tau) = 1$, cell size at division can be related to κ and k_P, yielding

$$1 = \frac{k_P}{\mu_X + \kappa}(V_d - V_0 2^{-\mu_X/\kappa}) \, , \tag{S.8}$$

where the cell volume at division is $V_d = V_0 e^{\kappa\tau}$. In the limit $\kappa >> \mu_X$, we arrive at

$$\Delta = \frac{\kappa}{k_P} \, , \tag{S.9}$$

where $\Delta = V_d - V_0$ is the added volume per generation. Because k_P and κ are both constant for a given growth medium, cells exhibit an adder mechanism in which a constant volume is added each generation regardless of birth size. In the opposite limit, in which $\kappa << \mu_X$, we get

$$V_d = \frac{\mu_X}{k_P} \, . \tag{S.10}$$

In slow-growing media, cells divide at a constant volume, and thus break from the adder mechanism and exhibit sizer behavior, in which cells divide at a set volume, regardless of birth volume.

Equation (S.8) can be rearranged to give the birth size as a function growth rate, such that

$$V_0 = \frac{\kappa + \mu_X}{k_P(2 - 2^{-\mu_X/\kappa})} \, . \tag{S.11}$$

As k_P is also a function of growth rate, Eq. (27) can be used to modify the equation above so that the dependency of V_0 on κ is fully realized. As a result we obtain

$$V_0 = \frac{\kappa + \mu_X}{\gamma(\alpha(\Delta\phi - \frac{\kappa + \mu_{\text{ns}}}{\kappa_t}) + \beta)(\kappa + \mu_{\text{ns}})(2 - 2^{-\mu_X/\kappa})} \, . \tag{S.12}$$

Outside of slow growing conditions, the effects of protein degradation are negligible. Assuming $\kappa \gg \mu_{\text{ns}}$ and $\kappa \gg \mu_X$, the above equation simplifies to

$$V_0 = \frac{1}{\gamma\alpha(\Delta\phi - \kappa/\kappa_t) + \gamma\beta} \, . \tag{S.13}$$

This expression asymptotically approaches a maximum growth rate given by $\kappa_{\max} = \kappa_t(\Delta\phi + \beta/\alpha)$. It is important to note that this theoretical maximum is nonphysical as it assumes that $f_X = \phi_X = 0$, which is never the case given our definition of f_X. The actually maximum growth rate occurs when $\phi_R = \phi_R^{\max}$, thus giving an upper limit to physical growth rate at $\kappa_{\max} = \kappa_t\Delta\phi$. Eq. (S.13) also predicts that there is no maximum cell size. However, our expression for f_X constrains cell size to a finite value. When allocation to ribosomes is maximal, $\phi_X = \beta$, such that the maximum birth volume V_0 is given by $V_0^{\max} = 1/\gamma\beta$.

SUPPLEMENTARY NOTE 5: ALTERNATIVE ALLOCATION STRATEGIES

To further probe how the specific underlying resource allocation strategy impacts size and division control, two alternative strategies were simulated for f_X. First a constant allocation strategy was simulated, in which f_X in invariant to nutrient perturbations. The degradation rate, μ_X, was left as a fitting parameter to ensure that our previous choice of μ_X was not responsible for disagreements with data, and the value of f_X was calculated such that the model would reproduce the initial steady-state added volume and interdivision time measurements. This yielded the parameters $\gamma f_X^{\text{const}} = 0.897$ and $\mu_X = 0$. As can be seen in Figures 3b-d in the main text, this allocation strategy fails to predict the overshoot in interdivision time following upshift, and incorrectly predicts a constant added volume regardless of nutrient environment. This behavior can be understood from Eq. (S.9), where now $k_P = \gamma f_X^{\text{const}}\kappa$, yielding $\Delta = V_0 = 1/\gamma f_X^{\text{const}}$. Thus, the added volume is no longer dependent on growth rate.

In addition, an allocation strategy in which f_X is proportional to f_R was also simulated. Specifically, $f_X^R = \alpha f_R + \beta$, where our previously calculated value for β was reused and α was calculated to reproduce the initial steady-state added volume and interdivision time measurements. μ_X was again left as a fitting parameter, yielding $\gamma\alpha = 0.656$, $\gamma\beta = 0.656$, and $\mu_X = 0$. As can be seen in Figures 3b-d in the main text, this allocation strategy fails to predict the overshoot in interdivision time following upshift, and incorrectly predicts a decrease in added volume with an increase in nutrient quality. This behavior again can be understood from Eq. (S.9), where now $k_P(\kappa) = \gamma f_X^R\kappa$, yielding $\Delta(\kappa) = V_0(\kappa) = 1/(\gamma\alpha(\kappa/\kappa_t + \phi_R^{\min}) + \gamma\beta)$ at steady-state. Thus, this allocation strategy predicts a decrease in cell size with increasing growth rate.

SUPPLEMENTARY NOTE 6: ANALYTICAL APPROXIMATION OF RELATIONSHIP BETWEEN STARVATION TIME AND GROWTH RATE RECOVERY TIME

Here we derive an analytical approximation relating the starvation time, τ_{starve}, to the time required to fully recovery the pre-starvation growth rate, τ_{recovery}. Specifically, we consider a scenario in which cells

growing at steady-state experience sudden starvation of duration τ_{starve}, followed by growth rescue via sudden nutrient exposure of identical quality to the initial growth conditions (Supplementary Figure 4a). We start by remembering the coupled equations which govern bacterial growth rate in time-varying nutrient environments,

$$\frac{\mathrm{d}a}{\mathrm{d}t} = \kappa_n(a,c)(\phi_R^{\max} - \phi_R) - \kappa_t(a)(\phi_R - \phi_R^{\min}) + \mu_{\text{ns}} , \tag{S.14}$$

$$\frac{\mathrm{d}\phi_R}{\mathrm{d}t} = \kappa_t(a)(\phi_R - \phi_R^{\min})(f_R(a) - \phi_R) , \tag{S.15}$$

where the growth rate is given by $\kappa = \mathrm{d}\ln M/\mathrm{d}t = \kappa_t(a)(\phi_R - \phi_R^{\min}) - \mu_{\text{ns}}$. To understand how the starvation time is related to recovery time, we must first understand how recovery time is affected by the macromolecular composition of the cell during starvation. If growth is rescued in starved cells by a step-wise increase in extracellular nutrients, there is an immediate influx of amino acids (a) which drives both translational efficiency and ribosomal allocation fraction close to their maximal values, such that $\kappa_t(a) \approx \kappa_t^0$ and $f_R(a) \approx \phi_R^{\max}$. Furthermore, Eq. (S.15) can be modified to express the dynamics in terms of the active ribosome fraction, $\phi_R^{\text{act}} = \phi_R - \phi_R^{\min}$, yielding

$$\frac{\mathrm{d}\phi_R^{\text{act}}}{\mathrm{d}t} = \kappa_t^0 \phi_R^{\text{act}}(\Delta\phi - \phi_R^{\text{act}}) , \tag{S.16}$$

where $\Delta\phi = \phi_R^{\max} - \phi_R^{\min}$. Thus immediately after upshift at $t = 0$, Eq. (S.15) is uncoupled from Eq. (S.14) and a closed-form expression can be obtained. Specifically,

$$\int_0^{\tau_{\text{recovery}}} \kappa_t^0 \mathrm{d}t = \int_{\phi_R^p}^{\phi_R^r} \frac{\mathrm{d}\phi_R^{\text{act}}}{\phi_R^{\text{act}}(\Delta\phi - \phi_R^{\text{act}})} , \tag{S.17}$$

$$\tau_{\text{recovery}} = \frac{1}{\kappa_t^0 \Delta\phi} \ln \frac{\phi_R^r(\phi_R^p - \Delta\phi)}{\phi_R^p(\phi_R^r - \Delta\phi)} , \tag{S.18}$$

where ϕ_R^p is the active ribosome fraction in the nutrient-poor condition immediately before upshift, and ϕ_R^r is the active ribosome fraction in the nutrient-rich condition at steady-state. Although ϕ_R^r is a steady-state quantity which remains the same before and after starvation if the nutrient quality is identical, ϕ_R^p is dependent on the starvation time, τ_{starve}. Similar to rescue, if the onset of starvation is step-wise such that $\kappa_n(0) = 0$, then immediately following downshift the dynamics of a are $\mathrm{d}a/\mathrm{d}t = \mu_{\text{ns}} - \kappa_t(a)\phi_R^{\text{act}}$. The regulatory functions of $\kappa_t(a)$ and $\kappa_n(a)$ ensure that the translational and nutritional fluxes are quickly balanced following nutrient perturbations. Consequently, the relaxation progresses along the a nullcline (Supplementary Figure 7c,d), allowing the dynamics of a to be eliminated, yielding the relationship

$$\mu_{\text{ns}} = \kappa_t(a)\phi_R^{\text{act}} . \tag{S.19}$$

Immediately following downshift, there is sudden depletion of amino acids which drives the ribosome allocation fraction close to its minimal value, such that $f_R(a) \approx \phi_R^{\min}$. Thus using Eqs. (S.15) and (S.19),

the dynamics of ϕ_R^{act} immediately following starvation are then

$$\frac{\mathrm{d}\phi_R^{\mathrm{act}}}{\mathrm{d}t} = -\mu_{\mathrm{ns}}\phi_R^{\mathrm{act}} \,. \tag{S.20}$$

To obtain the dependence of ϕ_R^p on τ_{starve}, we integrate Eq. (S.20) forward in time, yielding

$$\int_{\phi_R^r}^{\phi_R^p} \frac{\mathrm{d}\phi_R^{\mathrm{act}}}{\phi_R^{\mathrm{act}}} = \int_0^{\tau_{\mathrm{starve}}} -\mu_{\mathrm{ns}}\mathrm{d}t \,, \tag{S.21}$$

$$\phi_R^p = \phi_R^r e^{-\mu_{\mathrm{ns}}\tau_{\mathrm{starve}}} \,. \tag{S.22}$$

Finally, to elucidate the relationship between τ_{starve} and τ_{recovery}, we combine Eqs. (S.18) and (S.22) to obtain

$$\tau_{\mathrm{recovery}} = \frac{1}{\kappa_t^0 \Delta\phi} \ln \frac{\phi_R^r - \Delta\phi e^{\mu_{\mathrm{ns}}\tau_{\mathrm{starve}}}}{\phi_R^r - \Delta\phi} \,. \tag{S.23}$$

The growth rate can be approximated as $\kappa_r \approx \kappa_t^0 \phi_R^r - \mu_{\mathrm{ns}}$ in the nutrient-rich environment, allowing the recovery time to be related to the pre(post)-starvation growth rate,

$$\tau_{\mathrm{recovery}} = \frac{1}{\kappa_t^0 \Delta\phi} \ln \frac{\kappa_t^0 \Delta\phi e^{\mu_{\mathrm{ns}}\tau_{\mathrm{starve}}} - \kappa_r - \mu_{\mathrm{ns}}}{\kappa_t^0 \Delta\phi - \kappa_r - \mu_{\mathrm{ns}}} \,. \tag{S.24}$$

On physiologically relevant timescales, this analytical approximation explains the simulated behavior well (Supplementary Figure 4b).

Supplementary References

[1] M. Mori, Z. Zhang, A. Banaei-Esfahani, J. Lalanne, H. Okano, B. C. Collins, A. Schmidt, O. T. Schubert, D. Lee, G. Li, R. Aebersold, T. Hwa, and C. Ludwig, Molecular Systems Biology **17** (2021), 10.15252/msb.20209536.

[2] D. W. Erickson, S. J. Schink, V. Patsalo, J. R. Williamson, U. Gerland, and T. Hwa, Nature **551**, 119 (2017).

[3] M. Panlilio, J. Grilli, G. Tallarico, I. Iuliani, B. Sclavi, P. Cicuta, and M. C. Lagomarsino, Proceedings of the National Academy of Sciences **118** (2021), 10.1073/PNAS.2016391118.

[4] S. Taheri-Araghi, S. Bradde, J. T. Sauls, N. S. Hill, P. A. Levin, J. Paulsson, M. Vergassola, and S. Jun, Current Biology **25**, 385 (2015).

[5] Y. K. Kohanim, D. Levi, G. Jona, B. D. Towbin, A. Bren, U. A. Correspondence, and U. Alon, Cell Reports **23** (2018), 10.1016/j.celrep.2018.05.007.

[6] K. Sekar, R. Rusconi, J. T. Sauls, T. Fuhrer, E. Noor, J. Nguyen, V. I. Fernandez, M. F. Buffing, M. Berney, S. Jun, R. Stocker, and U. Sauer, Molecular Systems Biology **14**, e8623 (2018).

[7] F. Si, G. Le Treut, J. T. Sauls, S. Vadia, P. A. Levin, and S. Jun, Current Biology **29**, 1760 (2019).

[8] M. Scott, C. W. Gunderson, E. M. Mateescu, Z. Zhang, and T. Hwa, Science **330**, 1099 (2010).

[9] A. Schmidt, K. Kochanowski, S. Vedelaar, E. Ahrné, B. Volkmer, L. Callipo, K. Knoops, M. Bauer, R. Aebersold, and M. Heinemann, Nature Biotechnology **34**, 104 (2016).

[10] R. Phillips, J. Kondev, J. A. Theriot, H. G. Garcia, and N. Orme, *Physical Biology of the Cell (2nd ed.)* (Garland Science, 1998).
